# AP1-mediated reprogramming of EGFR expression triggers resistance to BLU-667 and LOXO-292 in RET-rearranged tumors

**DOI:** 10.1186/s13046-025-03392-w

**Published:** 2025-05-22

**Authors:** Daniela Esposito, Claudia Maria Ascione, Stefania Belli, Fabiana Napolitano, Alberto Servetto, Felice Pepe, Umberto Malapelle, Antonino Iaccarino, Giancarlo Troncone, Diletta Barone, Emilio Bria, Roberto Ferrara, Daniele Lorenzini, Giuseppe Lo Russo, Maria Rosa Ghigna, Arianna Marinello, Mihaela Aldea, Benjamin Besse, Luigi Formisano, Roberto Bianco

**Affiliations:** 1 Department of Clinical Medicine and Surgery, University of Naples “Federico II”, Naples, 80131 Italy; 2 Department of Public Health, University of Naples Federico II, Naples, Italy; 3 University Cattolica del Sacro Cuore, Fondazione Policlinico Universitario Agostino Gemelli, IRCCS, Rome, Italy; 4 Isola Tiberina Hospital, Gemelli Isola, Rome, Italy; 5 University Vita-Salute San Raffaele, Milan, Italy; 6 Department of Advanced Diagnostics, Fondazione IRCCS Istituto Nazionale Dei Tumori, Milan, Lombardia Italy; 7 Medical Oncology Department 1, Fondazione IRCCS Istituto Nazionale Dei Tumori, Milan, Italy; 8 Department of Pathology, Gustave Roussy, Villejuif, France; 9 Department of Cancer Medicine, Gustave Roussy, Villejuif, France; 10 Paris-Saclay University, Paris, France

**Keywords:** Non-small cell lung cancer, RET, RET inhibitors, EGFR, Drug resistance, Combination targeted therapy

## Abstract

**Background:**

Non-small cell lung cancer (NSCLC) is a significant global health challenge, with 2% of cases fuelled by *RET* rearrangements. RET inhibitors (RETi) have revolutionized treatment for these patients, but resistance remains an important clinical challenge limiting therapy effectiveness. This study investigated the mechanisms underlying resistance to RETi.

**Methods:**

NSCLC cells were exposed to increasing doses of RETi (pralsetinib/BLU-667 and selpercatinib/LOXO-292) to generate resistant cells. RNA-Sequencing analysis identified differentially expressed genes in resistant *versus* sensitive cells, followed by in vitro and in vivo functional assays to explore novel therapeutic strategies. Additionally, tumor biopsies from *RET*-rearranged NSCLC patients who exhibited cancer progression on RET inhibitor therapy were analyzed.

**Results:**

RNA-sequencing analysis revealed the upregulation of the EGFR signaling pathway and hyperactivation of AP1 complex members in resistant cells compared to sensitive cells. Silencing of *EGFR* and AP1 complex members significantly reversed drug resistance, whereas *EGFR* overexpression reduced the sensitivity of parental Lc2/AD cells to RET inhibitors. Furthermore, the combination of RET and EGFR inhibitors showed synergistic antitumor activity in vitro and hindered tumor growth in mouse models with resistant cell xenografts. Notably, we observed a significant increase in EGFR expression in tumor biopsies from NSCLC patients treated with RET inhibitors who experienced disease progression, further validating the clinical relevance of our findings.

**Conclusions:**

This study elucidates EGFR's role in mediating resistance to RET inhibitors in NSCLC patients. These findings offer insights into therapeutic adaptation and explore personalized combinations of RET and EGFR inhibitors for improved clinical outcomes.

**Supplementary Information:**

The online version contains supplementary material available at 10.1186/s13046-025-03392-w.

## Introduction

Non–small cell lung cancer (NSCLC) is one of the leading causes of cancer-related deaths worldwide [1]. Among the subtypes of NSCLC, adenocarcinoma is undergoing a significant surge, representing approximately 50% of all cases [2]. Approximately 2% of adenocarcinoma NSCLCs harbor *RET* (REarranged during Transfection) rearrangements [3]. RET oncogene is activated primarily through chromosomal inversion or translocation, leading to the fusion of the Ret C-terminal kinase domain with the N-terminal portion of an unrelated protein [4]. The most common *RET* fusion partners in lung cancer are *KIF5B* (70–90%) and *CCDC6* (10–25%) [5–7]. Fusions result in chimeric proteins with intact kinase domains, determining constitutive activation for ligand-independent dimerization and phosphorylation. Consequently, this event enhances Ret downstream signaling pathways, including mitogen-activated protein kinase (MAPK), extracellular signal-regulated kinase (ERK), phosphatidylinositide 3-kinase (PI3 K), and c-Jun N-terminal kinase (JNK) [8, 9]. *RET* genomic alterations in NSCLC disrupt the physiological activation of proliferating and apoptotic pathways as well as differentiation and chemotaxis, ultimately leading to uncontrolled cell growth [10].

The Food and Drug Administration (FDA) and the European Medicines Agency (EMA) recently accelerated the approval of selpercatinib/LOXO- 292 and pralsetinib/BLU- 667 for adult patients with advanced *RET* fusion-positive NSCLC, marking significant progress in targeted therapy. These approvals were based on promising results from the LIBRETTO- 001 (NCT03157128) and ARROW (NCT03037385) clinical trials [11–19]. Despite positive results, not all patients benefit from these treatments, and almost all of them eventually progress. While on-target resistance mutations are identified in only 10–15% of cases, the majority of resistance mechanisms seem to involve off-target pathways, such as the activation of bypass receptor tyrosine kinases [20]. Furthermore, nearly half of the patients exhibited no identifiable genomic resistance mechanisms, limiting the efficacy of new-generation RET inhibitors to a specific patient subset. Therefore, the development of novel therapeutic strategies to circumvent RET inhibitor resistance is an unmet clinical need.

Herein, we report the results of our study, in which we identified hyperactivation of EGFR signaling as a potential mechanism of resistance to RET inhibition. We demonstrated that EGFR hyperactivation, due to aberrant regulation of AP1 complex members, drives a proliferative phenotype in *RET* fusion-positive NSCLC cells resistant to BLU-667 and LOXO-292. Notably, our preclinical data revealed that targeting EGFR resensitized resistant cells and xenografts to RET inhibition. These findings were further supported by the observation of increased EGFR expression in patients with *RET* rearrangement who progressed to RET inhibitor therapy. These results provide a scientific rationale supporting the combined use of RET and EGFR inhibitors, potentially delaying the emergence of resistance and overcoming it when it arises.

## Methods

### Cell lines and inhibitors

The human non-small cell lung cancer cell line Lc2/AD (harboring *CCDC6-RET* fusion) was purchased from the American Type Culture Collection (ATCC, Manassas, VA, USA) and maintained in ATCC recommended media (RPMI- 1640:F12 = 1:1) supplemented with 10% fetal bovine serum (FBS) (Gibco, Waltham, MA, USA) and 1 × antibiotic/antimycotic (Gibco). The human papillary thyroid cancer cell line TPC- 1 (harboring *CCDC6-RET* fusion) was kindly provided by Prof. Massimo Santoro (Medicina Molecolare e Biotecnologie Mediche, Università degli Studi di Napoli Federico II, Napoli, Italy). The cells were maintained in DMEM supplemented with 10% FBS (Gibco, Waltham, MA, USA) and 1 × antibiotic/antimycotic (Gibco). All the cell lines used in this study were maintained at 37 °C in a humidified atmosphere of 5% CO2 in air. Drug-resistant cells (BluR and LoxoR) were generated after 6 months of treatment with increasing doses of BLU- 667 (Pralsetinib-SelleckChem-Houston, TX, USA; #S8716) and LOXO- 292 (Selpercatinib; SelleckChem-Houston, TX, USA; #S8781), with final concentrations reaching up to 500 nM. All the cell lines were tested for mycoplasma contamination and authenticated via short tandem repeat (STR) profiling by BMR Genomics. All experiments on these cell lines were performed between the 4 th and 16 th passages.

### Clonogenic and dose‒response assays

 For clonogenic assays, sensitive and drug-resistant cells (5 × 10^4^/well) were seeded in triplicate in 6-well plates in complete media. After 24 h, the cells were treated with vehicle (DMSO), BLU-667, LOXO-292 (final concentration of 500 nM), afatinib (SelleckChem, Houston, TX, USA; #S1011; final concentration of 50 nM), osimertinib (SelleckChem, Houston, TX, USA; # S7297; final concentration of 200 nM) or erlotinib (SelleckChem, Houston, TX, USA; # S7786; final concentration of 1 µM). The media were replenished every 3 days until the control wells reached 70–80% confluency. The monolayers were then fixed and stained with 20% methanol/80% water/0.25% crystal violet for 20 min, washed with water, and dried. Then, the plates were scanned in Brother MFCL2710DW (Brother) at 300 dpi, the stained cells were solubilized with 20% acetic acid solution, and the absorbance was quantified via spectrophotometric detection at 590 nm using a plate reader (GloMax® Discover Microplate Reader, Promega).

For the dose‒response assays, parental and drug-resistant cell lines were seeded in 96-well plates, and after 24 h, the cells were treated with DMSO or increasing concentrations of BLU-667 or LOXO-292 (12 doses ranging from 0.1 nM to 10 μM, threefold dilution). Six days later, a CellTiter-Glo Luminescent Cell Viability Assay (Promega; #G8461) was performed. An equal volume of CellTiter-Glo reagent was added to the culture medium in each well. After 2 min on an orbital shaker to induce cell lysis, the absorbance was quantified by spectrophotometric detection using a plate reader (GloMax® Discover Microplate Reader (Promega).

To test the synergy between BLU-667/LOXO-292 and afatinib, cells were treated with increasing doses of each drug alone or in combination every 72 h until the vehicle-treated controls reached ∼90% confluence. The combination indices were determined using the Chou‒Talalay method based on the median-effect equation. The resulting combination index (CI) offers a quantitative definition of the additive effect (CI = 1), synergism (CI < 1), and antagonism (CI > 1) of drug combinations.

### Immunoblot analysis

The cells were washed with PBS and lysed with radioimmunoprecipitation assay (RIPA) buffer (#sc-24948, Santa Cruz, USA) according to the manufacturer’s instructions. Snap-frozen tumor fragments were homogenized using a TissueLyser (Qiagen) and lysed in RIPA lysis buffer. The lysates were kept on ice for 30 min, vortexed, and then centrifuged at 13,400 rpm for 20 min at 4 °C. The protein concentrations in the supernatants were quantified using a BCA protein assay kit (Pierce™; #23,225). Total protein (20 μg) was separated on bis–tris 3–8% gradient gels (NuPAGE) and transferred to nitrocellulose membranes using the Trans-Blot® Turbo™ RTA Mini Nitrocellulose Transfer Kit (Bio-Rad, Hercules, CA, USA). The membranes were blocked with 5% nonfat dry milk at room temperature for 45 min, followed by overnight incubation with primary antibodies at 4 °C. All the following antibodies were purchased from Cell Signaling Technology or Santa Cruz Biotechnology: EGFR (1:1000 - #sc- 1005); phospho-EGFR (Tyr1068- 1:1000 #2234); ERK2 (1:1000- #sc- 1647); phospho-p44/42 MAPK (Erk1/2) (Thr202/Tyr204 - 1:1000- #9101); FosB (1:1000- #2251); Fra1 (1:1000- #sc- 28310); Ret (1:500- #3220); phospho-Ret (Tyr905- 1:500- #3221); Shc (1:500- #2432); phospho-Shc (Tyr239/240- #2434); and α/β Tubulin (1:1000- #2148). The nitrocellulose membranes were washed and incubated with HRP-conjugated anti-rabbit or anti-mouse secondary antibodies for 45 min at room temperature. Immunoreactive proteins were visualized by enhanced chemiluminescence using SuperSignal West Pico PLUS Chemiluminescent Substrate (Thermo Fisher Scientific, Waltham, MA, USA). The films were imaged using Brother MFCL2710DW (Brother) at 300 dpi. Densitometric analysis was conducted using ImageJ 1.54 d software (NIH, Bethesda, MD, USA), and results are presented as fold change (FC). Intensity values were normalized to the expression signals of loading controls.

### Lung cancer spheroid assay

The Lc-2/ad, BluR, and LoxoR cell lines were seeded in quadruplicate into Ultra-Low attachment 96 plates (#CLS3474, Corning) at a density of 5 × 10^3^ cells per well in 100 µL of 10% RPMI- 1640:F12. After 48 h, images were captured (T0) using an InvitrogenTM EVOSTM FL imaging system (20X magnification). The spheroids were treated with the indicated drugs for 72 h. The area of the spheroids was quantified using ImageJ 1.53 software (NIH, Bethesda, MD, USA) and normalized to the T0 area. Spheroids transfected with siCTRL and si*EGFR* were established 48 h after siRNA transfection. They were then treated, and the areas were measured as described above.

### siRNA transfection

Small interfering RNAs (siRNAs) targeting *EGFR* (#226,558,293; #226,558,296; #226,558,299 hs.Ri. EGFR), *FOSL1* (#229,852,154; #229,852,157; #229,852,160; hs.Ri. FOSL1), and *FOSB* (#233,819,949; #233,819,952; #233,819,955; hs.Ri. FOSB) were purchased from Integrated DNA Technology. The cells were transfected with 50 nM siRNA via Lipofectamine RNAiMAX® transfection reagent (Thermo Fisher Scientific) following the manufacturer’s instructions. Forty-eight hours post-transfection, the cells were seeded in medium supplemented with 10% FBS in ultralow attachment 96-well plates (5 × 10^3^/well for spheroid assays), 60-mm plates (for immunoblot analysis), or 6-well plates for the clonogenic assay or cell count (5 × 10^4^/well) and treated with the indicated drug concentrations.

### Plasmid overexpression

Human pLenti-C-mGFP control (PS100071 V) and mGFP-tagged-EGFR (RC217384L4 V) lenti-ORF particles were purchased from OriGene. In Lc2/ad parental cells, lenti-ORF particles (10^7 TU/mL) were transfected with 8 µg/mL polybrene (Sigma‒Aldrich) to generate stably transduced cell lines. After 48 h of incubation, the transduced cells were selected with 1 µg/mL puromycin, and the cell culture media was changed every 72 h.

### Cell cycle analysis by propidium iodide staining

Lc-2/ad, BluR, and LoxoR cells were seeded in 100-mm plates. The cells were incubated in serum-free media for 24 h and then treated with media containing 10% FBS + the indicated drugs for 24 h. Next, the cells were washed with PBS and fixed in 99% methanol for 3 h at − 20 °C. The cells were then incubated with FxCycle™ PI/RNase Staining Solution (Invitrogen™) following the manufacturer’s instructions. Fluorescence-activated cell sorting (FACS) analysis was performed usingvia a FACSVerse™ (BD Biosciences).

### Immunofluorescence assay

2 × 10^4^ Lc-2/ad, BluR, and LoxoR were seeded in sterile chamber slides (Nunc Lab-Tek) for 24 h and treated with BLU-667 and LOXO-292 for 24 h. The cells were fixed in paraformaldehyde and 4% in 1X PBS for 30 min. Fixed cells were washed with 1X PBS and then incubated in blocking buffer (0.5% BSA in 1X PBS) for 45 min, followed by an overnight incubation with an anti-EGFR rabbit polyclonal antibody (1:200; #sc- 1005) at 4 °C. The chamber slides were washed with 1X PBS and incubated with a donkey anti-rabbit IgG (H + L) cross-reaction secondary antibody, Cy™2 (1:200; Jackson ImmunoResearch; #711–225 - 152), for 1 h. Fluorescent mounting medium with DAPI was used as the mounting solution (Abcam; #104,139). Images were collected using a fully automated inverted microscope.

### ChIP q‒PCR analysis

BluR cells were cultured in 100-mm dishes and incubated with serum-free RPMI- 1640:F12 medium containing 1% formaldehyde for 10 min at room temperature. Crosslinking was quenched using glycine. The plates were subsequently washed three times with ice-cold 1X PBS three times, and the cells were scraped off the plate with ice-cold PBS containing Halt™ Protease Inhibitor Cocktail (Thermo Scientific™- Catalog: # 87,786). The cells were subsequently centrifuged at 720 × g for 10 min at 4 °C. The pellets were subsequently resuspended in 1.5 mL of sonication buffer (1% SDS, 10 mmol/L EDTA, 50 mmol/L Tris–HCl, pH 8.0–8.1) supplemented with protease inhibitors. Chromatin was sonicated via a Bioruptor. Sonicated chromatin was adjusted to a final concentration of 200 mM NaCl and incubated at 65 °C overnight. The following day, the chromatin was incubated with RNase A for 30 min at 37 °C, followed by incubation with proteinase K for 1 h at 42 °C. DNA was purified via a QIAquick PCR Purification Kit (Cat 28,106). DNA shearing was checked, with an average fragment size of 200–750 bp. To conduct chromatin immunoprecipitation (ChIP), the sonicated chromatin was eluted with ChIP dilution buffer (0.01% SDS buffer, 1.1% Triton X- 100, 1.2 mmol/L EDTA, 167 mmol/L NaCl, 16.7 mmol/L Tris–HCl, pH 8.0–8.1) with protease inhibitors and precleared with Gammabind G Sepharose beads (GE Healthcare, Cat no. 17088501) previously washed three times with ChIP-dilution buffer and blocked in 0.5% BSA at 4 °C for 1 h. The precleared chromatin was incubated at 4 °C overnight with primary antibody. The following day, Gammabind G Sepharose beads were added to the antibody pulldowns for 2 h at 4 °C. Next, the beads were washed once in Buffer I (0.1% SDS, 1% Triton X- 100, 2 mmol/L EDTA, 20 mmol/L Tris–HCl, 150 mmol/L NaCl, and protease inhibitors, pH 8.0–8.1), once in Buffer II (0.1% SDS, 1% Triton X- 100, 2 mmol/L EDTA, 20 mmol/L Tris–HCl, 500 mmol/L NaCl, and protease inhibitors), once in Buffer III (0.25 M LiCl, 1% NP40, 1% Na-deoxycholate, 1 mmol/L EDTA, 10 mmol/L Tris–HCl, pH 8.0–8.1) and twice in TE buffer, pH 8.0–8.1. The complexes were eluted from the beads in elution buffer (0.1 M NaHCO3, 1% SDS) at 65 °C for 10 min twice in a thermomixer (Chromatin Immunoprecipitation (ChIP) Assay Kit- Catalog #17–295). Eluates from the antibody pulldowns and sonicated chromatin (as input controls) were adjusted to 200 mmol/L NaCl and incubated at 65 °C overnight. The following day, the DNA was purified using a QIAquick PCR Purification Kit (QIAGEN; Catalog #28,104). The ChIP products and input DNA were analyzed in triplicate by quantitative PCR (qPCR) using iTaq Universal SYBR Green Supermix and a CFX Connect Real-Time PCR Detection System (Bio-Rad). Actinβ was used as a housekeeping gene for data normalization, and relative gene expression was measured using the 2^−ΔΔ^ Ct method. qPCR oligo pairs for the *EGFR* promoter based on Cistrome BD data were analyzed in the UCSC genome browser: 5’- ATGACTCTGCTTGGTGCAGAA- 3’ (Fwd); 5’- TCACACTTGAGTCTTGGCCCC- 3’ (Rev); 5’- ACCCATGGCTGGTTGCAATA- 3’ (Fwd); 5’- AGAGCCAGCGTCGGATAATG- 3’ (Rev); 5’- CCTGGCCAAAGGGTCATCAT- 3’ (Fwd); and 5’- CTGTCTCACCCAGTCCACAC- 3’ (Rev).

### Oncomine tumor mutation load assay

Each sample was tested using The Oncomine Tumor Mutational Load Assay (Thermo Fisher Scientific) on the Ion Torrent S5 GS NGS platform (Thermo Fisher Scientific) according to the manufacturer’s instructions. This panel was able to evaluate single-nucleotide variants, copy number variations, and insertions/deletions in 409 cancer-related genes (a complete gene list is available at https://www.thermofisher.com/order/catalog/product/A3790 9#/A37909). Briefly, 10 ng of DNA from each sample was manually amplified, according to the manufacturer’s instructions. The libraries were then diluted to 30 ng and pooled for sequencing analysis. The pool was automatically loaded onto the Ion 540 chip according to the manufacturer’s instructions. Sequencing data were analyzed using Ion Reporter Suite software (v.5.0.2.1; Thermo Fisher Scientific) using a customized workflow. Data analysis was performed as follows: variants with ≥ 5 × allele coverage and a quality score ≥ 20 within an amplicon coverage of at least 500 × alleles were called. Finally, the frequency of each mutant allele was recorded.

### RNA-Sequencing and gene set enrichment analysis

Parental and drug-resistant cells were seeded in triplicate in 100-mm dishes and treated with DMSO, BLU-667, or LOXO-292. After 24 h, the cells were harvested, and RNA was purified using the RNeasy Plus Kit (QIAGEN; #74,134). A Qubit fluorimeter was used to determine the sample concentration. Genewiz (Germany) conducted library preparation, RNA-sequencing analysis, and reading quality control. Gene set enrichment analysis (GSEA) was performed on normalized data using GSEA_4.3.2. Connectivity map (https://clue.io/about), DepMap (https://depmap.org/portal/interactive/), and Integrated Motif Activity Response Analysis (ISMARA; https://ismara.unibas.ch/mara/) were conducted using online tools.

### Xenograft studies

All animal procedures were performed in accordance with the institutional guidelines of the University of Naples Animal Care Committee and the Declaration of Helsinki. BluR cells (7 × 10^6^ cells per mouse) were suspended in PBS and Matrigel (Corning #356,234) at a 1:1 ratio and injected subcutaneously into the right flank of 6-week-old female Balb/c (nu +/nu +) mice (Charles River). Approximately 4 weeks after tumor cell injection, mice bearing tumors measuring ≥ 200 mm3 were randomized (five per group) to treatment with 1) vehicle (5% DMSO, 40% PEG300, 5% Tween, and 50% ddH₂O, by oral gavage); 2) BLU- 667 (10 mg/kg b.i.d., by oral gavage); 3) afatinib (20 mg/kg, q.d. by oral gavage); and 4) BLU-667 plus afatinib, for 15 days. Animal weights and tumor diameters (measured with calipers) were measured twice weekly, and the tumor volume in mm^3^ was calculated using the following formula: volume = width2 × length/2. At the end of the treatment, the tumors were harvested and snap-frozen in liquid nitrogen before total protein extraction and immunoblot analysis.

### Patient cohort and immunohistochemistry (IHC)

Patient biopsies for immunohistochemistry analysis were obtained from Gustave Roussy (Paris), IRCCS (Rome) and IRCCS (Milan). Pre-treatment biopsies were collected within six months before starting selpercatinib. Post-treatment biopsies were obtained at primary or metastatic sites after that progression disease (PD) was assessed. For paired biopsies (pre- and post- selpercatinib), two 4-μm thick sections from each tissue block were cut at the microtome: one section was attached to a normal slide and stained with hematoxylin/eosin for adequacy evaluation, and one section was attached to positively charged slides for subsequent IHC. Soon after the sections were cut, the slides were placed in an oven at 48 °C for 1 h and then stored at room temperature until the next day.

IHC staining was performed using an automated staining system (BenchMark XT; Ventana Medical Systems, Inc., Arizona, USA) using the UltraView Universal DAB Detection Kit. The standard IHC protocol was used, and pre-diluted anti-EGFR (DAKO EGFR pharmDx kit for Gustave Roussy samples and polyclonal anti-epidermal growth factor receptor/EGFR/ErbB1 (Quartet-ref. 2-EP040 - 10 – biocyc GmbH & Co. KG) antibodies were used for IRCCS (Rome and Milan) samples at 37 °C for 32 min. Briefly, after deparaffinization and rehydration, the paraffin-embedded tissue sections were blocked with 3% hydrogen peroxide for 4 min at room temperature. Antigen retrieval was performed on the selected samples using CC1 solution (Ventana Medical Systems, Inc., Arizona, USA) containing EDTA (pH 8.0) at 95 °C for 64 min. The tissue sections were then incubated with UltraView Universal HRP Multimer and developed with 3,3′-diaminobenzidine (DAB) for 4 min. Counterstaining was performed with hematoxylin for 8 min and a bluing reagent for 4 min.

EGFR expression was assessed by an expert pathologist, who assigned values ranging from 0 (no staining or membranous staining in < 10% of tumor cells) to 1/2/3 (incomplete, weak/moderate, and strong and complete staining in > 10% of tumor cells) using the H score (0–300).

### Statistical analysis

Statistical analyses were performed using GraphPad Prism software, version 10.2 (San Diego, California, USA). Data were expressed as mean ± standard deviation (SD). For comparisons between two groups, Student’s t-test was used, while two-way analysis of variance (ANOVA) was applied for comparisons among three or more groups. For RNA-seq data comparisons, Wilcoxon test was utilized. Correlations were analyzed using Pearson’s correlation analysis. A p-value of less than 0.05 was considered statistically significant.

## Results

### Generation and characterization of CCDC6-RET NSCLC cell lines resistant to BLU-667 and LOXO-292

**Fig. 1:**
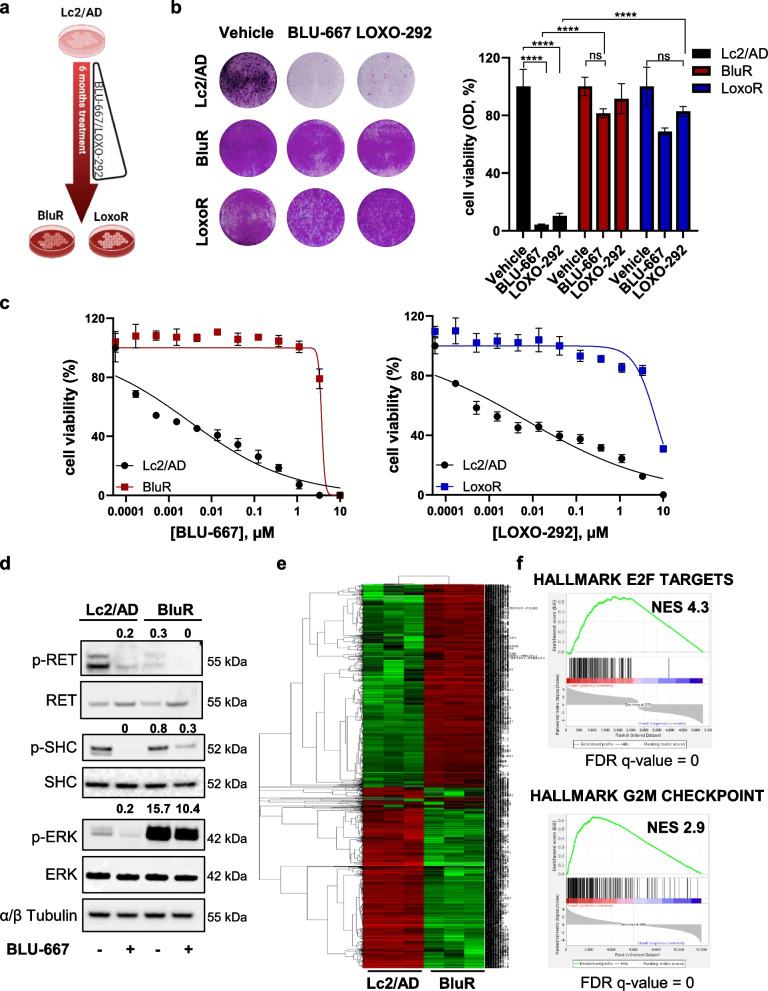
*CCDC6-RET* NSCLC cells resistant to BLU-667 and LOXO-292. **a** Schematic representation illustrating the generation of BLU-667-resistant (BluR) and LOXO-292-resistant (LoxoR) Lc2/AD cells. **b** Left, images of crystal violet-stained monolayers of Lc2/AD, BluR, and LoxoR cells seeded in 6-well plates and treated with vehicle, BLU-667 (500 nM) or LOXO-292 (500 nM). Right, bar graph showing quantification by spectrophotometric detection of the integrated intensity values as % of vehicle-treated controls in Lc2/AD (black), BluR (red), and LoxoR (blue) cells (ns *p* > 0.05, ****p < 0.0001; 2way ANOVA Bonferroni’s multiple comparisons). **c** Twelve-point dose–response curves of parental Lc2/AD (black line) and BluR (red line), and Lc2/AD (black line) and LoxoR (blu line) cells treated with BLU-667 (left) or LOXO-292 (right). After 6 days of treatment, cells were lysed and quantified with ATP cell titer Glo. Each data point represents the percentage of cell viability relative to vehicle-treated controls. **d** Immunoblots of Lc2/AD parental and BluR cells treated with BLU-667 (500 nM). After treatment for 4 h, whole-cell lysates were prepared and subjected to immunoblot analyses with the indicated antibodies. Numbers above blots indicate FC variations compared to vehicle-treated Lc2/AD cells. Images are representatives from three independent experiments. **e** Heatmap showing unsupervised hierarchical clustering of differentially expressed genes (FDR < 0.01) in BluR *versus* Lc2/AD parental cells upon BLU-667 treatment for 24 h. Up- and down-regulated genes are represented in red and green, respectively. **f** Gene set enrichment analysis (GSEA) of significantly de-regulated pathways in BluR cells *versus* Lc2/AD cells shown with Normalized Enriched Score (NES) and FDR q-value. For all panels, data are expressed as mean ± standard deviation (SD) of three separate experiments, indicated by error bars

To investigate the signaling pathways that could sustain resistance to RET inhibition, we generated NSCLC cells resistant to the drugs BLU-667/pralsetinib and LOXO-292/selpercatinib, herein defined as BluR and LoxoR cells, respectively. Briefly, Lc2/AD NSCLC cells (harboring the *CCDC6-RET* fusion) were chronically exposed to increasing doses of BLU-667 or LOXO-292 (Fig. 1a). After 6 months, resistant cells grew in the presence of 500 nM of either compound, whereas in parental cells the treatment completely abrogated both monolayer growth and spheroid formation (Fig. 1b, Supplementary Fig. 1a, left and right panels). Moreover, dose‒response proliferation assays confirmed that the BluR and LoxoR cells were less responsive to each drug than the Lc2/AD parental cells (Fig. 1c, left and right panels; Suppl. Fig. 1b‒c). To further support these findings, cell cycle analysis demonstrated that, in response to RET inhibitors (RETi), Lc-2/AD-sensitive cells showed an increased percentage of cells in the G1 phase and a decreased percentage of cells in the G2/M phase. In contrast, BluR and LoxoR cells showed a defective G1 phase increase, underlying the acquisition of resistance to RETi (Supplementary Fig. 1d-e).

To assess whether the insensitivity of resistant cells to RET inhibitors resulted from newly acquired mutations in *RET* oncogene or in other known oncogenic drivers in NSCLC (such as *MET* or KRAS, already known as mechanisms of resistance to RETi [21]), we conducted a mutational screening for 409 cancer-related gene abnormalities. Interestingly, molecular alterations in LC2/AD (i.e., *KMT2D*, *COL1 A1*, and *PARP1*) were mirrored in BluR cells (Suppl. Fig. 2), ruling out the possibility that new mutations in *RET* oncogene or other driver genes in BluR cells could be responsible for drug resistance.

Hence, we hypothesized that the acquisition of resistance to BLU-667 and LOXO-292 could be sustained by the activation of alternative signaling pathways, as previously reported for other tyrosine kinase inhibitors [22–29]. Immunoblotting suggested that RETi continued to impair Ret activation in BluR and LoxoR cells, as indicated by the effects on p-Ret and p-SHC (Fig. 1d, Supplementary Fig. 3a). However, upon chronic treatment with RETi, BluR and LoxoR cells showed sustained phospho-ERK (an effector of mitogen-activated protein kinase (MAPK)) (Fig. 1d, Supplementary Fig. 3a), despite the presence of BLU-667 and LOXO-292.

Thus, to investigate the transcriptional reprogramming sustaining resistance to RET inhibition, RNA-Sequencing of BluR, LoxoR, and parental Lc-2/AD cells (± BLU-667 or ± LOXO-292 for 24 h) was performed. Heatmaps revealed the peculiar transcriptional signatures of BluR and LoxoR cells compared with the Lc2/AD cells (Fig. 1e, Supplementary Fig. 3b). Gene set enrichment analysis (GSEA; [30]) of the differentially regulated genes in the BluR and LoxoR cells revealed hyperexpression of pro-proliferative signaling pathways, such as E2F-responsive and G2-M checkpoint genes, which was consistent with the enhanced survival capabilities of the resistant cells compared with the sensitive parental cells (Fig. 1f, Supplementary Fig. 3c).

### EGFR hyperactivation promotes resistance to RET inhibitors

**Fig. 2:**
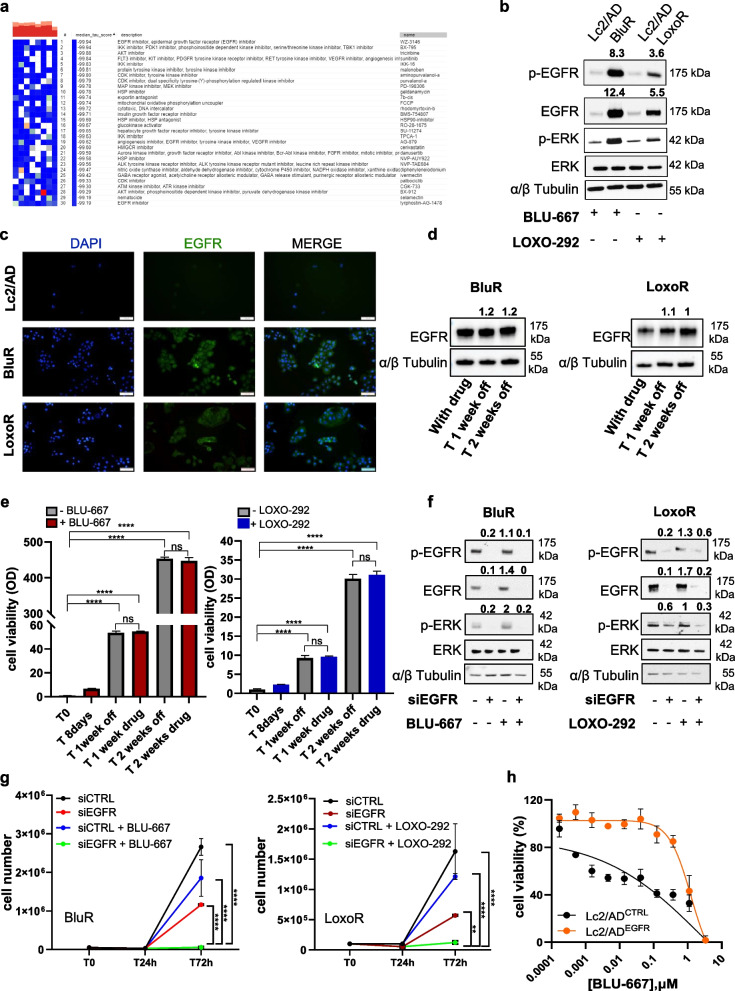
EGFR expression and activation regulates sensitivity to RET inhibitors. **a. **Connectivity map analysis to identify drugs that could potentially reverse the expression of resistance-associated genes. **b** Immunoblots of Lc2/AD parental, BluR and LoxoR cells treated with BLU-667 (500 nM) or LOXO-292 (500 nM). After treatment for 24 h, whole-cell lysates were prepared and subjected to immunoblot analyses with the indicated antibodies. Numbers above blots indicate FC variations compared to their counterpart Lc2/AD cells, treated with BLU-667 or LOXO-292. Images are representatives from three independent experiments. **c** Immunofluorescence staining of EGFR (Cy2) in Lc2/AD, BluR, and LoxoR cells. Nuclei were counterstained in blue (DAPI) (magnification 20 ×). **d** Immunoblots of BluR (left) and LoxoR (right) cells treated with BLU-667 (500 nM) or LOXO-292 (500 nM) for 8 days and then washed out for 1 and 2 weeks. Whole-cell lysates were prepared and subjected to immunoblot analyses with the indicated antibodies. Numbers above blots indicate FC variations compared to cells under treatment for 8 days. Images are representatives from three independent experiments. **e** Bar graph showing quantification by spectrophotometric detection of the integrated intensity values as % of T0, in BluR (left) and LoxoR (right). Cells were treated with the drugs for 1 week and then allowed to grow in media containing the respective drugs (BLU-667 in red and LOXO- 292 in blu) or without drugs (gray). (**p < 0.01; ****p < 0.0001; 2way ANOVA Bonferroni’s multiple comparisons). **f** Immunoblots of BluR (left) and LoxoR (right) cells knocked down for scramble or *EGFR* siRNAs and treated or not with BLU-667 and LOXO-292, respectively. After treatment for 72 h, whole-cell lysates were prepared and subjected to immunoblot analyses with the indicated antibodies. Numbers above blots indicate FC variations compared to cells transfected with scramble siRNAs. Images are representatives from three independent experiments. (**p < 0.01; ****p < 0.0001; 2way ANOVA Bonferroni's multiple comparison). **g** Line chart indicating cell number of BluR (left panel) and LoxoR (right panel) cells treated with scramble small interfering RNAs (siCTRL) or siRNAs targeting *EGFR* (si*EGFR*) in the presence or not of BLU-667 and LOXO-292, respectively. **h** Twelve-point dose–response curves of Lc2/AD over-expressing GFP control vector (pLenti-C-mGFP-P2 A-Puro; Lc2/AD^CTRL^, black line) or *EGFR* over-expressing cells (Lc2/AD^EGFR^, orange line) treated with BLU-667. After 6 days of treatment, cells were lysed and quantified with ATP cell titer Glo. Each data point represents the percentage of cell viability relative to vehicle-treated controls. For all panels, each point represents the mean ± SD from three independent experiments

We sought to identify potential deregulated pathways that might promote resistance to RET inhibitors. Leveraging RNA-Sequencing data, we conducted connectivity map analysis to determine which pathway inhibition could most effectively reverse the resistant phenotype. The top 150 upregulated genes in RETi-resistant cells were selected according to the protocol, which generated a list of drugs capable of eliciting a gene expression pattern opposite to the observed signature [30]. Interestingly, EGFR pathway inhibitors emerged as the top candidates for reversing the transcriptional signature associated with resistance to RET inhibitors, with the most negative median score correlating with the best drug (Fig. 2a). This result prompted us to investigate the effects of EGFR inhibition in resistant models.

Hence, we first evaluated EGFR expression in cell lines resistant to both RET inhibitors using immunoblot and immunofluorescence assays. Notably, we observed an increase in EGFR expression and activation, as well as an increase in p-ERK levels, in BluR and LoxoR cells (Fig. 2b-c). Hence, we hypothesized that EGFR hyperactivation could trigger compensatory mechanisms to RET inhibition, in turn activating MAPK signaling.

Then, we investigated whether the transcriptional reprogramming sustaining resistance was maintained after the withdrawal of the drugs. Interestingly, we found that BluR and LoxoR cells, following the removal of BLU-667 and LOXO-292 for 2 weeks, respectively, exhibited sustained EGFR levels (Fig. 2d, left and right panels) and growth profiles comparable to those of cells continuously exposed to the drugs (Fig. 2e, left and right panels).

Hence, to elucidate the causal role of EGFR in conferring resistance to RET inhibitors, we explored the effects of *EGFR* silencing on BluR and LoxoR cell growth. We observed that the combination of *EGFR* silencing and RET inhibition led to decrease in ERK activation (Fig. 2f) and a drastic and significant reduction in cell viability and spheroid formation (Fig. 2g, Suppl. Fig. 4a). Notably, *EGFR* knockdown alone did not elicit any effects on Lc-2/AD parental cells (Suppl. Figure 4b-c). Conversely, we stably overexpressed *EGFR* in Lc- 2/AD cells (Lc- 2/AD^EGFR^) (Suppl. Figure 4d). Compared with the Lc- 2/AD control (Lc- 2/AD^CTRL^), Lc- 2/AD^EGFR^ exhibited reduced sensitivity to BLU-667 and LOXO-292 associated with ERK activation (Fig. 2h, Suppl. Figure 4e-f).

Furthermore, since Lc2/AD represents the only available model harboring RET rearrangement for NSCLC, we employed TPC1 cells, a papillary thyroid cancer (PTC) cell line carrying *CCDC6-RET* fusion. *RET* fusions occur in 10–20% of patients with PTC, qualifying these patients for treatment with RET inhibitors. Thus, we aimed to evaluate whether EGFR signaling plays a similar role in conferring resistance to RET inhibitors in this model. Surprisingly, we observed a lower sensitivity to BLU-667 and LOXO-292 in TPC1 cells (Suppl. Figure 5a) compared with Lc2/AD cells. Interestingly, we observed early increases in EGFR and p-ERK levels upon RETi treatment (Suppl. Figure 5b, left and right panels). Notably, concurrent silencing of *EGFR* and treatment of cells with either BLU-667 or LOXO-292 significantly impaired TPC1 viability, unlike RET inhibition or *EGFR* knockdown (Suppl. Figure 5c, left and right panels).

### Concurrent pharmacologic inhibition of RET and EGFR restores BLU-667 and LOXO-292 sensitivity in vitro and in vivo

**Fig. 3:**
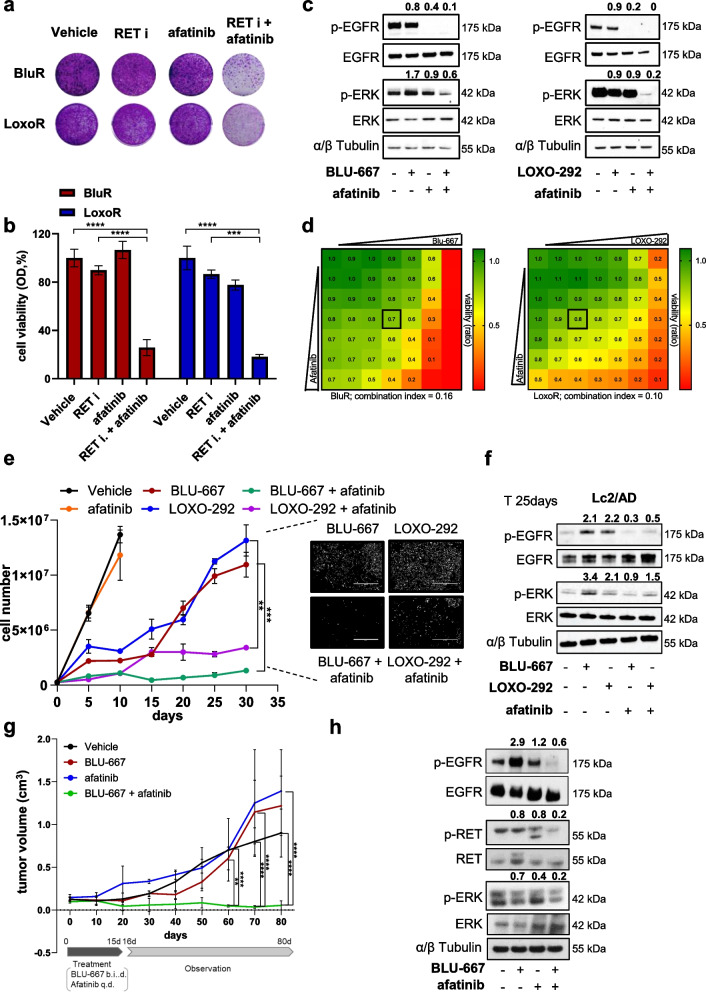
The combination of RET and EGFR inhibitors impairs tumor growth in vitro and in vivo. **a** Images of crystal violet-stained monolayers of BluR and LoxoR cells seeded in 6-well plates and treated with vehicle, BLU-667 (500 nM) or LOXO-292 (500 nM) (RET i), afatinib (50 nM), and the combination (RET i + afatinib). **b** Bar graph showing quantification by spectrophotometric detection of the integrated intensity values as % of vehicle-treated controls in BluR (red) and LoxoR (blue) cells. (***p < 0.001; ****p < 0.0001; 2way ANOVA Bonferroni’s multiple comparisons). **c** Immunoblots of BluR (left) and LoxoR (right) cells treated with BLU-667 or LOXO-292 ± afatinib (left and right panels, respectively). Whole-cell lysates were prepared and subjected to immunoblot analysis with the indicated antibodies. Numbers above blots indicate FC variations compared to vehicle-treated cells. Images are representative of three independent experiments. **d** Viability assay to test the synergy between BLU-667 (left) or LOXO-292 (right) and afatinib in BluR and LoxoR cells, respectively. Cells were treated with increasing concentrations of each drug alone or with both drugs every 72 h until the vehicle-treated controls reached ~ 90% confluency. Cell monolayers were then lysed with ATP cell titer Glo reagent and the number of viable cells was determined based on the quantification of ATP, indicating metabolically active cells. Combination indices were determined using the Chou-Talalay test. Numbers inside each box indicate the ratio of viable cells to untreated cells from three independent experiments. **e** Line chart indicating the number of Lc2/AD cells treated with vehicle, BLU-667 (500 nM), LOXO-292 (500 nM), afatinib (50 nM), and their combinations for 30 days. (**p < 0.01, ***p < 0.001; 2way ANOVA Bonferroni’s multiple comparisons).Representative images of cells treated with BLU-667 ± afatinib and LOXO-292 ± afatinib for 30 days. **f** Immunoblots of Lc2/AD cells treated with BLU-667, LOXO-292, afatinib and the combination for 25 days. Whole-cell lysates were prepared and subjected to immunoblot analysis with the indicated antibodies. Numbers above blots indicate FC variations compared to vehicle-treated cells. Images are representative of three independent experiments. **g** BluR xenografts established in Balb/c mice. Once tumors reached ≥ 200 mm3, the mice were randomized to treatment with vehicle, BLU-667 (10 mg/kg b.i.d.), afatinib (20 mg/kg q.d.), or both drugs. The BluR tumor growth was monitored for 3 months. Each data point represents the mean tumor volume in cm^3^ ± SEM (*n* =5 mice per arm). **i** Whole-cell lysates were prepared from proteins extracted from BluR xenografts after four days of treatment and subjected to immunoblot analyses with the indicated antibodies. Numbers above blots indicate FC variations compared to vehicle-treated tumors. Images are representative of three independent experiments. For all panels, each point represents the mean ± SD from three independent experiments

Based on these results, we tested the effects of combining EGFR inhibitors with either BLU-667 or LOXO-292 on RETi-resistant cells. EGFR is pharmacologically inhibited by afatinib, a clinically approved irreversible EGFR inhibitor. Interestingly, afatinib led to a significant reduction in cell viability when combined with BLU- 667 or LOXO- 292 in BluR and LoxoR cells (Fig. 3a-b). Single-agent treatments did not affect proliferation or ERK activation, but concurrent treatment with BLU-667/LOXO-292 and afatinib resulted in robust blockade of MAPK signaling and viability (Fig. 3a-c). These findings were further validated using the first-generation EGFR inhibitor erlotinib and the third-generation EGFR inhibitor osimertinib, which significantly resensitized BluR and LoxoR cells to RET inhibitors (Supplementary Fig. 6a-f). Furthermore, to verify whether the combinations of BLU-667/afatinib and LOXO-292/afatinib had synergistic or additive effects, we assessed drug interactions via the Chou‒Talalay method [31]. Combination studies using increasing concentrations of BLU-667 or LOXO-292 and afatinib demonstrated striking synergy between these agents in inhibiting the growth of BluR and LoxoR cells (combination index, CI < 1 indicates synergy; BluR CI = 0.16; LoxoR CI = 0.10; Fig. 3d).

Similar to the NSCLC BluR and LoxoR models, concomitant treatment with RET inhibitors and afatinib significantly decreased TPC1 cell viability (Suppl. Figure 7a, left and right panels). Impairment of cell viability was associated with a reduction in p-ERK (Suppl. Figure 7b, left and right panels). Finally, we also evaluated whether BLU-667/LOXO-292 had a synergistic effect with afatinib via the Chou‒Talalay method and we observed notable synergism between RET and EGFR inhibitors (BLU-667/afatinib CI = 0.40; LOXO-292/afatinib CI = 0.1, Suppl. Figure 7c, left and right panels), indicating a crucial role of EGFR in TPC1 sensitivity to RETi.

Next, we investigated whether combined RET/EGFR inhibition could delay the development of drug resistance. After 25 days of treatment with the single agents BLU-667 or LOXO-292, cells began to proliferate again, whereas no regrowth was observed under combinatory treatment with afatinib (Fig. 3e) This regrowth following RET inhibitors treatment may be driven by EGFR activation, which is suppressed by the combination therapy (Fig. 3f). Moreover, upon removal of inhibitors, cells began to proliferate and recolonize the wells with higher regrowth in cells treated with BLU-667 or LOXO-292 alone compared to the growth rates observed in cells that received combination therapy (Suppl. Fig. 8a).

Finally, we tested the safety and efficacy of combining afatinib with BLU-667 in vivo. BluR xenografts in Balb/c nude mice revealed that treatment with BLU-667 or afatinib alone had no effects on tumor growth, whereas the combination significantly affected tumor growth (Fig. 3g). Importantly, the combined treatment did not negatively affect the body weights of the mice (Suppl. Fig. 8b). Western blot analysis of tumors treated with afatinib* plus* BLU-667 revealed downregulation of p-ERK levels (Fig. 3h), which was consistent with the in vitro findings.

### AP1 complex members modulate resistance to selective RET inhibitors regulating EGFR expression

**Fig. 4:**
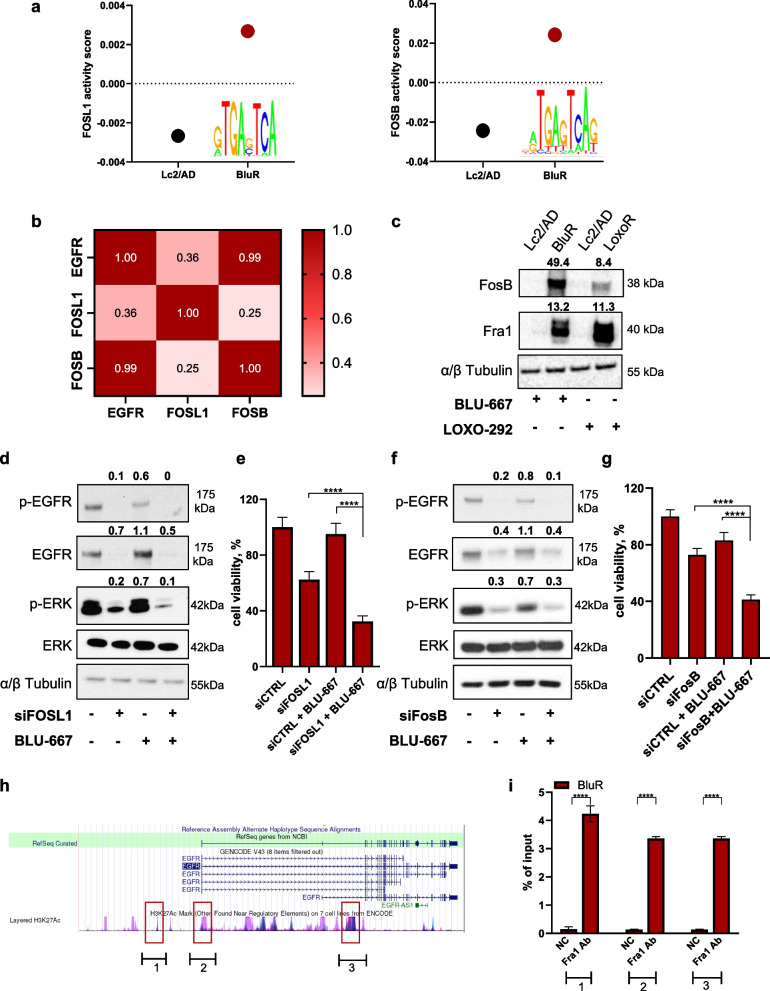
AP1 complex members regulate *EGFR* expression in cells resistant to RET inhibitors. **a** Graph showing activity score of transcriptional factor *FOSL1* (left) and *FOSB* (right) in Lc2/AD and BluR cells based on RNA-Sequencing data using Integrated System for Motif Activity Response Analysis (ISMARA). **b** Schematic representation of Pearson correlation for expression levels of *EGFR*, *FOSL1* and *FOSB* genes, based on our RNA-Sequencing data. **c** Immunoblots of Lc2/AD and BluR cells treated with BLU-667 (500 nM) and Lc2/AD and LoxoR cells treated with LOXO-292 (500 nM) for 24 h. Whole-cell lysates were prepared and subjected to immunoblot analyses with the indicated antibodies. Numbers above blots indicate FC variations compared to their counterpart Lc2/AD cells, treated with BLU-667 or LOXO-292. Images are representatives from three independent experiments. **d**,** f** Immunoblots of BluR cells knocked down for scramble (siCTRL) or *FOSL1* (si*FOSL1; *d) or *FOSB* (si*FOSB*; f) siRNAs and treated or not with BLU-667. After treatment for 72 h, whole-cell lysates were prepared and subjected to immunoblot analyses with the indicated antibodies. Numbers above blots indicate FC variations compared with cells transfected with scramble siRNAs. Images are representatives from three independent experiments.** e**, **g** Bar graph showing cell viability of BluR cells transfected with siRNAs against *FOSL1* (e) and *FOSB* (g) and treated with BLU-667 (500 nM). Data are plotted as a percentage of vehicle-treated cells transfected with siRNAs scramble (siCTRL) (****p < 0.0001; 2way ANOVA). **h** Schematic representation of the genomic *locus* encompassing *EGFR* gene. In the top part the exon/intron structure. In the bottom part, ENCODE tracks for H3K27Ac mapping across promoter and introns of *EGFR* are represented as coverage curves (peaks). **i** ChIP-qPCR with a Fra1 antibody or normal rabbit IgG (control), at the *EGFR* regulator regions. Enrichment values are expressed as percent (%) of input. (****p < 0.0001; Student's t-test). For all panels, each point represents the mean ± SD from three independent experiments

We aimed to uncover the biological mechanisms underlying *EGFR* overexpression in BluR and LoxoR cells, which are ultimately responsible for resistance to RETi. We interrogated our RNA-Sequencing data to determine putative transcription factors regulating *EGFR* expression in resistant cells by using the Integrated System for Motif Activity Response Analysis (ISMARA) [32]. By cross-referencing the top activated transcription factors obtained by ISMARA with known *EGFR* regulators, we identified Fra1 (encoded by *FOSL1*) and FosB (encoded by *FOSB*), both well-known members of the AP1 complex, as the most activated factors in resistant cells (Fig. 4a). Notably, interrogating the available omics datasets in DepMap portal, we found that *FOSL1* and *FOSB* expression positively correlated with *EGFR* expression in different cancer types (Suppl. Figure 9a-b; Pearson coefficients of 0.281 and 0.597, respectively), including lung cancer (Suppl. Figure 9c-d; Pearson coefficients of 0.647 and 0.290, respectively). The positive correlation between *FOSL1/FOSB* and *EGFR* was further confirmed by our RNA-Sequencing data (Fig. 4b). Thus, we verified the expression of these two transcription factors by western blot analysis, which revealed higher expression of Fra1 and FosB in BluR and LoxoR cells than in Lc2/AD cells (Fig. 4c). We subsequently knocked down *FOSL1* and *FOSB* in BluR and LoxoR cells to verify whether they could recapitulate the effects of *EGFR* silencing. Both si*FOSL1* and si*FOSB* reduced *EGFR* expression and activation (Fig. 4d, 4f), further confirming their roles in regulating *EGFR*. Moreover, the combination of either si*FOSL1* or si*FOSB* with BLU- 667 or LOXO-292 significantly decreased BluR and LoxoR viability (Fig. 4e, 4 g; Suppl. Figure 10a-b). Conversely, similar to the events occurring upon *EGFR* knockdown, in sensitive Lc2/AD cells, si*FOSL1* and si*FOSB* did not elicit any additive effects on BLU-667 or LOXO-292 treatment, considering both cell count and spheroid growth analysis (Suppl. Figure 10c-f).

Finally, to determine the regulatory role of AP1 complex members in *EGFR* expression, we performed ChIP‒qPCR analysis to investigate whether direct binding of Fra1 or FosB occurs in *EGFR* regulatory regions. For this purpose, we first interrogated the Cistrome Data Browser to verify the putative binding regions of AP1 complex members on the *EGFR* promoter or enhancer regions. Based on the available Chip-Seq datasets, we selected eight candidate regions common to different databases as potentially bound by Fra1 (representative image in Fig. 4h; no data were available for FosB). Interestingly, three of the eight selected regions were amplified by qPCR following Fra1-selective chromatin immunoprecipitation (Fig. 4i). Conversely, chromatin immunoprecipitation using a specific anti-FosB antibody did not yield similar results. Hence, our analysis confirmed that Fra1, but not FosB, binds to *EGFR* regulatory regions and modulates its expression.

### EGFR levels modulation in NSCLC patients treated with RET inhibitors: clinical insights

Our preclinical data revealed that *EGFR* overexpression is a key event that underpins resistance to RET inhibition. Therefore, we examined the clinical relevance of EGFR as a resistance mechanism in a cohort of patients treated with RETi. For six patients diagnosed with *RET*-rearranged NSCLC treated with RET inhibitors, we found available matched biopsies, including a RETi-baseline biopsy (before the start of the RETi, no more than six months before strating treatment) and a biopsy collected at the time of progression from RETi (RETi-PD). For all patients, we examined EGFR protein levels by immunohistochemistry (IHC) at RETi-baseline and after progression biopsies. In four out of six patients, the findings align with our hypothesis.

In particular, Patient #1 was treated with selpercatinib after progressing to platinum + pemetrexed + bevacizumab as first-line treatment. After 6 months of RETi, the patient experienced pleural, adrenal, and bone progression. Similarly, patient #2, following progression after chemoimmunotherapy in combination with platinum + pemetrexed + pembrolizumab, started second-line treatment with selpercatinib and continued treatment for 6 months until the treatment was stopped for progression in the liver and peritoneum sites. After progressing on vinorelbine, Patient #3 was characterized as RET-rearranged and enrolled for selpercatinib treatment, which was administrated for eleven months. All three patients had *KIF5B-RET* fusion.

**Fig. 5:**
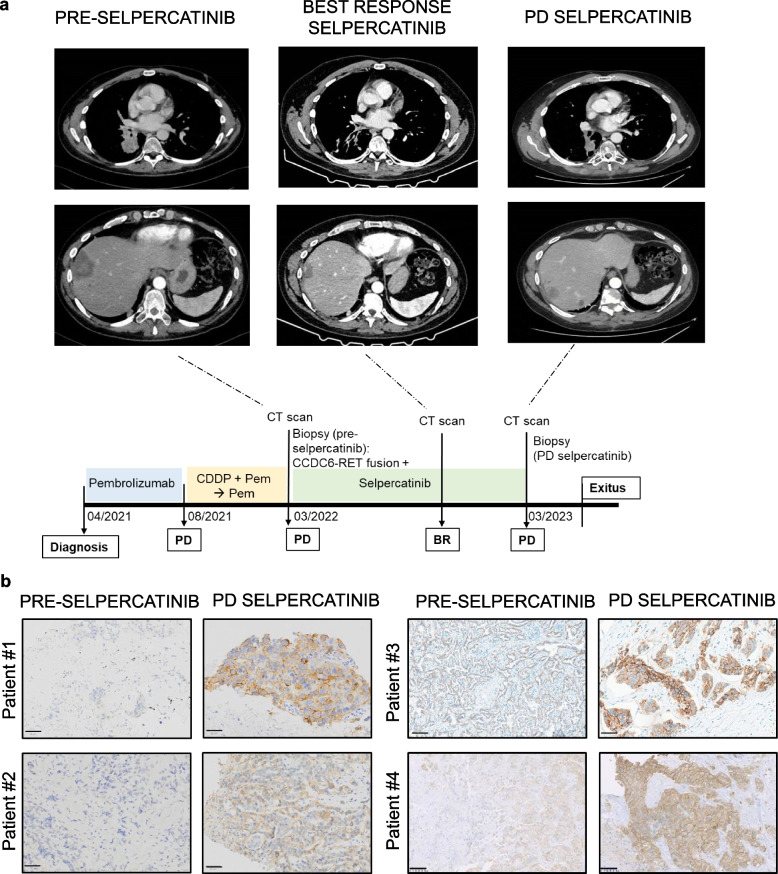
Immunohistochemistry analysis reveals increased EGFR expression in disease-progression tumor biopsies. **a** Contrast-enhanced computed-tomography images of the thorax (upper) and abdomen (bottom) were acquired at baseline (on the left), showing a mass in the inferior lobe of the right lung, and a liver metastasis with central low attenuation (compatible with necrosis) and enhanced margins. These target lesions are shown at best response (central, best response) and at progression after selpercatinib treatment (right), where new liver lesions appeared (bottom panel showing the new lesion). CDDP = Cisplatin, cis-diamminedichloroplatinum; Pem = pemetrexed; PD = progression disease; BR = best response **b** Representative images of tissue sections subjected to IHC for EGFR. For patient #1, representative images of lung (pre-selpercatinib) and adrenal (Progression disease, PD selpercatinib) sections; for patient #2, representative images of brain metastasis (pre-selpercatinib) and liver (PD selpercatinib); for patient #3, representative images of liver (pre-selpercatinib and PD selpercatinib); for patient #4, representative images of liver (pre-selpercatinib) and pelvis (PD selpercatinib); Scale bar 50 µm

At the time of diagnosis, patient #4 (with available representative computerized axial tomography; Fig. 5a) exhibited high (80%) PDL- 1 expression, but *RET* fusion status was not known (other molecular alterations investigated: wild-type *EGFR, BRAF,* and *KRAS*; no rearrangements in *ALK* and *ROS1*; tested negative for *MET* and *NTRK* alterations). After treatment with anti-PD- 1 pembrolizumab as the first-line therapy, the patient progressed after an initial clinical response. A biopsy of the liver metastasis indicated the presence of a *CCDC6-RET* fusion; therefore, the patient was administered the RET inhibitor selpercatinib as a subsequent intervention. Despite an initial partial response, after one year of treatment, the patient experienced disease progression with the appearance of new lung, pelvic, and liver secondary lesions (Fig. 5a). Biopsy of the new metastatic lesion in the pelvis was performed for comprehensive analysis.

Interestingly, we discovered that, compared with pre-selpercatinib biopsies, post-progression treatment tissues presented discernible increases in EGFR protein levels in four patients (three cases switching from absent/weak staining in pre-treatment samples to moderate/strong staining in post-treatment tissues and one case from moderate to strong; Fig. 5b). Hence, these clinical findings mirror the results of our preclinical models of RET resistance.

## Discussion

Over the past decade, molecular targeted therapies have become increasingly pivotal in the therapeutic landscape of oncogene-addicted cancers. Notably, pralsetinib and selpercatinib have demonstrated unprecedented efficacy in the treatment of patients with lung or thyroid tumors, driven by *RET* gene rearrangements. However, despite the remarkable responses in many cases, the emergence of resistance mechanisms poses significant challenges to the clinical efficacy of these therapies.

Clinical evidence has highlighted the reactivation of the MAPK pathway in selpercatinib-resistant tumors, driven by secondary *RET* mutations or alterations in other driver genes, such as *MET* amplification and *RAS* or *BRAF* mutations [21]. However, a well-documented occurrence in solid cancers is signaling rebound prompted by transcriptional reprogramming rather than gene alterations [24–26, 33, 34]. Hu et al. demonstrated in preclinical models that MAPK reactivation induced by treatment with BLU-667 led to the upregulation of Aurora A/B kinases, which resulted in tumor regression when inhibited in combination with RET inhibition [35].

Herein, we present evidence suggesting that transcriptional reprogramming, leading to *EGFR* overexpression and reactivation, rather than point mutations or copy number variations, may influence the response to RET inhibition. Our findings support the efficacy of combined targeting of RET and EGFR in *CCDC6-RET* NSCLC and thyroid cancer cells, successfully preventing tumor cell growth under RET-resistant conditions. In our NSCLC-resistant cell lines and xenograft models, targeting EGFR, in addition to RET inhibition, resensitized cells to anti-RET treatment. This effect was particularly pronounced in *RET*-rearranged papillary thyroid cancer cells, where early EGFR activation reduced sensitivity to RET inhibitors, and targeting EGFR with afatinib significantly enhanced the response. Notably, upfront combination therapy with RET and EGFR inhibitors prevents compensatory EGFR activation, which otherwise contributes to tumor regrowth and resistance to RET inhibitors. This highlights the therapeutic potential of dual inhibition in delaying or overcoming adaptive resistance mechanisms. Mechanistically, we found that in NSCLC cells, the transcription factor Fra1 regulates *EGFR* expression by directly binding to its genomic sequence, offering insights into the underlying mechanisms of *EGFR* overexpression in RET-resistant models. Fra1, encoded by *FOSL1*, is known to operate as a heterodimeric complex with other members of the activator protein- 1 (AP- 1) complex, such as *FosB*. High expression of Fra1 is closely associated with tumor cell proliferation, apoptosis, and migratory events, exerting oncogenic effects, and is correlated with advanced tumor stage and poor survival in non-small cell lung cancer patients [36]. Its overexpression has been described as a consequence of MAPK hyperactivation [37]. Although EGFR targeting did not modulate Fra1 expression (data not shown), gene knockdown of the AP1 complex members Fra1 and FosB modulated *EGFR* expression, demonstrating direct binding of Fra1 with *EGFR* regulatory regions. To the best of our knowledge, our findings are the first to shed light on the mechanisms underlying *EGFR* hyperactivation in RET-resistant models and propose strategies to overcome these events through a clinically feasible combination approach.

Notably, the preliminary clinical evidence supports our findings. Specifically, four out of six patients treated with selpercatinib exhibited increased *EGFR* expression upon disease progression, as evidenced by immunohistochemistry of the metastatic sites. Notably, although our preclinical findings were obtained in *CCDC6-RET* cell lines, we observed the same mechanism in patients carrying *CCDC6-RET* and the most common *KIF5B-RET* rearrangements. Furthermore, although small in number, the analysis of our cohort revealed that EGFR upregulation may represent a common resistance mechanism. These findings have substantial implications, suggesting the feasibility of using immunohistochemistry to assess *EGFR* expression levels to guide anti-EGFR therapy selection in patients with progressive disease.

In conclusion, our study underscores the importance of the combined targeting of RET and EGFR to overcome resistance mechanisms in RET-rearranged cancers. By elucidating the complex mechanisms underlying therapy adaptation, we provide valuable insights that could inform the development of novel therapeutic approaches aimed at preventing and overcoming the resistance mechanisms that currently limit the efficacy of RET inhibitors.

## Supplementary Information

Supplementary Material 1.

## Data Availability

The datasets used and/or analysed during the current study are available from the corresponding author on reasonable request.

## Abbreviations

NSCLC: Non-small cell lung cancer
RET: REarranged during Transfection
EGFR: Epidermal growth factor receptor
AP1: Activator protein 1
KIF5B: Kinesin family member 5B
CCDC6: Coiled-Coil Domain-Containing Protein 6
MAPK: Mitogen-activated protein kinase
ERK: Extracellular signal-regulated kinase
PI3K: Phosphatidylinositide 3-kinase
JNK: C-Jun N-terminal kinase
FDA: Food and Drug Administration
EMA: European Medicines Agency
ATCC: American Type Culture Collection
FBS: Fetal bovine serum
DMEM: Dulbecco's Modified Eagle Medium
CI: Combination index
PBS: Phosphate-buffered saline
RIPA: Radioimmunoprecipitation assay
BCA: Bicinchoninic acid assay
FosB: FBJ murine osteosarcoma viral oncogene homolog B
Fra1: Fos-related antigen 1
Shc: Src homology 2 domain containing
FOSL1: FOS-like 1
ORF: Open Reading Frame
GFP: Green Fluorescent Protein
FACS: Fluorescence-activated cell sorting
PI: Propidium Iodide
RNase: Ribonuclease
BSA: Bovine Serum Albumin
ChIP: Chromatin Immunoprecipitation
qPCR: Quantitative Polymerase Chain Reaction
EDTA: Ethylenediaminetetraacetic acid
HCl: Hydrochloric acid
mM: MilliMolar
SDS: Sodium Dodecyl Sulfate
Fwd: Forward
Rev: Reverse
NGS: Next-generation sequencing
GSEA: Gene set enrichment analysis
ISMARA: Integrated Motif Activity Response Analysis
DMSO: Dimethyl Sulfoxide
PEG: Polyethylene Glycol
BID: Bis in die
OS: Oral administration
PD: Progression disease
IHC: Immunohistochemistry
DAB: Diaminobenzidine
MET: Mesenchymal Epithelial Transition factor
KRAS: Kirsten rat sarcoma viral oncogene homolog
KMT2D: Lysine Methyltransferase 2D
COL1 A1: Collagen Type I Alpha 1
PARP1: Poly (ADP-Ribose) Polymerase 1
PTC: Papillary thyroid cancer
BRAF: B-Raf Proto-Oncogene, Serine/Threonine Kinase
ALK: Anaplastic Lymphoma Kinase
ROS1: ROS Proto-Oncogene 1, Receptor Tyrosine Kinase
NTRK: Neurotrophic Tyrosine Receptor Kinase
PD- 1: Programmed Cell Death Protein 1
