# Supplementary material for: AP1-mediated reprogramming of EGFR expression triggers resistance to BLU-667 and LOXO-292 in RET-rearranged tumors
Source: J Exp Clin Cancer Res. 2025 May 22;44:154. doi: 10.1186/s13046-025-03392-w (PMC12096543; doi:10.1186/s13046-025-03392-w)
Supplement: Supplementary file 1 — Supplementary Material 1. [file 13046_2025_3392_MOESM1_ESM.pdf]

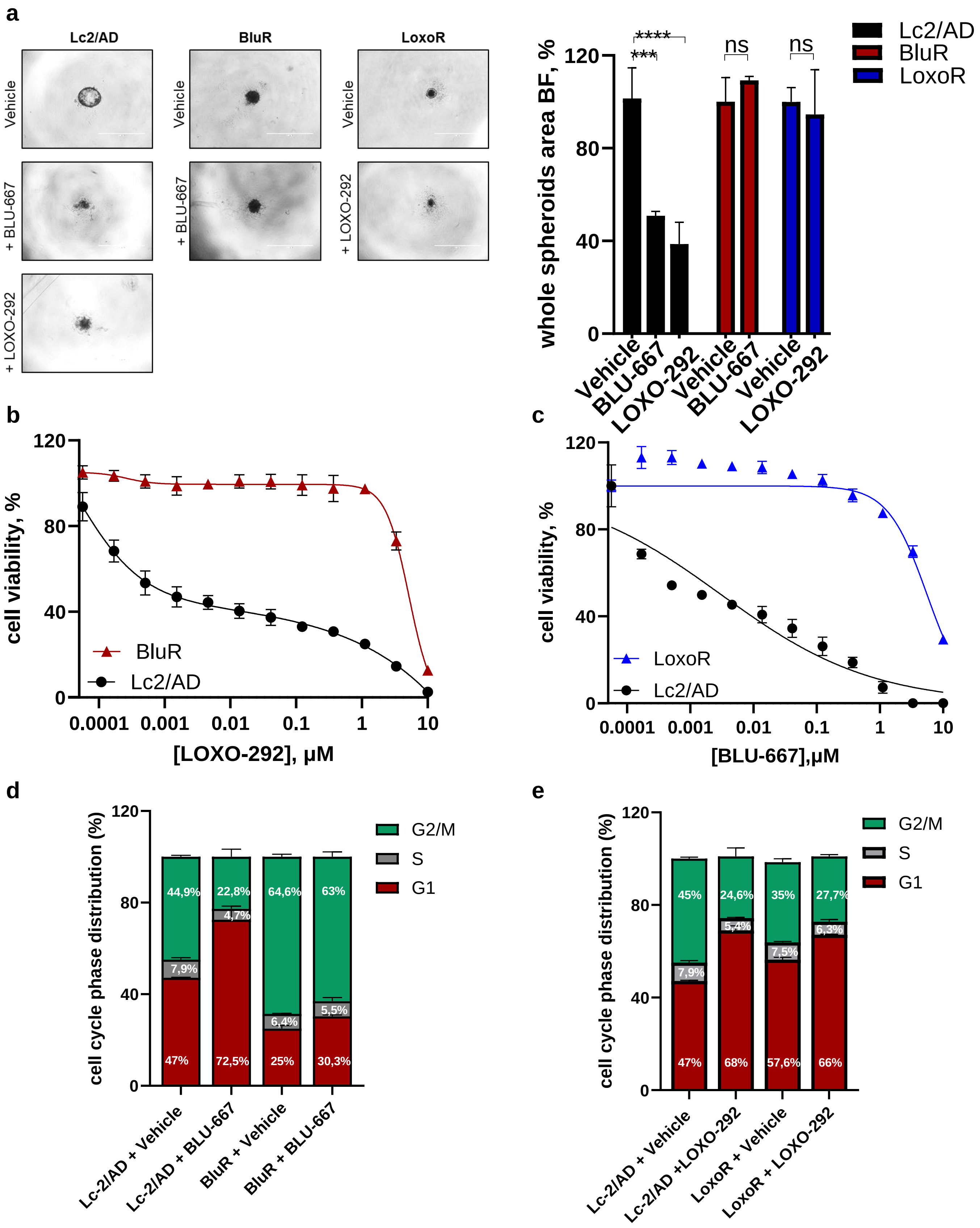

a
Lc2/AD
BluR
LoxoR
Vehicle
+ BLU-667
+ LOXO-292
whole spheroids area BF, %
****
***
ns
ns
Lc2/AD
BluR
LoxoR
Vehicle
BLU-667
LOXO-292
b
cell viability, %
BluR
Lc2/AD
[LOXO-292], µM
c
cell viability, %
LoxoR
Lc2/AD
[BLU-667],µM
d
cell cycle phase distribution (%)
G2/M
S
G1
44,9%
22,8%
64,6%
63%
7,9%
4,7%
6,4%
5,5%
47%
72,5%
25%
30,3%
Lc-2/AD + Vehicle
Lc-2/AD + BLU-667
BluR + Vehicle
BluR + BLU-667
e
cell cycle phase distribution (%)
G2/M
S
G1
45%
24,6%
35%
27,7%
7,9%
5,4%
7,5%
6,3%
47%
68%
57,6%
66%
Lc-2/AD + Vehicle
Lc-2/AD +LOXO-292
LoxoR + Vehicle
LoxoR + LOXO-292

Supplementary Fig1: BluR and LoxoR cells are cross-resistant to LOXO-292 and BLU-667, respectively . (a) Representative images of Lc2/AD or BluR and LoxoR spheroids cultured for 72 hours under BLU-667 (250nM) and LOXO-292 (250nM) treatments. All images were captured at 20x magnification (Bars = 200 μm) (left panel). Bar graph showing the whole spheroids plotted as a percentage relative to of vehicle-treated spheroids area (right panel) (*** $p < 0.001$ ****$p < 0.0001$; One-way ANOVA). **(b)** Twelve-point dose-response curves of parental Lc2/AD (black line) and BluR (red line) cells treated with LOXO-292, and **(c)** of Lc2/AD (black line) and LoxoR (blu line) cells treated with BLU-667. After 6 days of treatment, cells were lysed and quantified with ATP cell titer Glo. Each data point represents the percentage of cell viability relative to vehicle-treated controls. Each point represents the mean of viability ± SD from three independent experiments. **(d-e)** bar graphs indicating the percentage of Lc2/AD and BluR (d) and LC2/AD and LoxoR (e) cells in G0,G1 and S phases upon BLU-667 and LOXO-292 treatments, respectively. Cells were serum-starved for 24 h and then treated with vehicle (DMSO) or each of the indicated inhibitors (all at 500nM) containing media for 24 h. Following treatment, cells were stained with propidium iodide and analyzed by FACS.

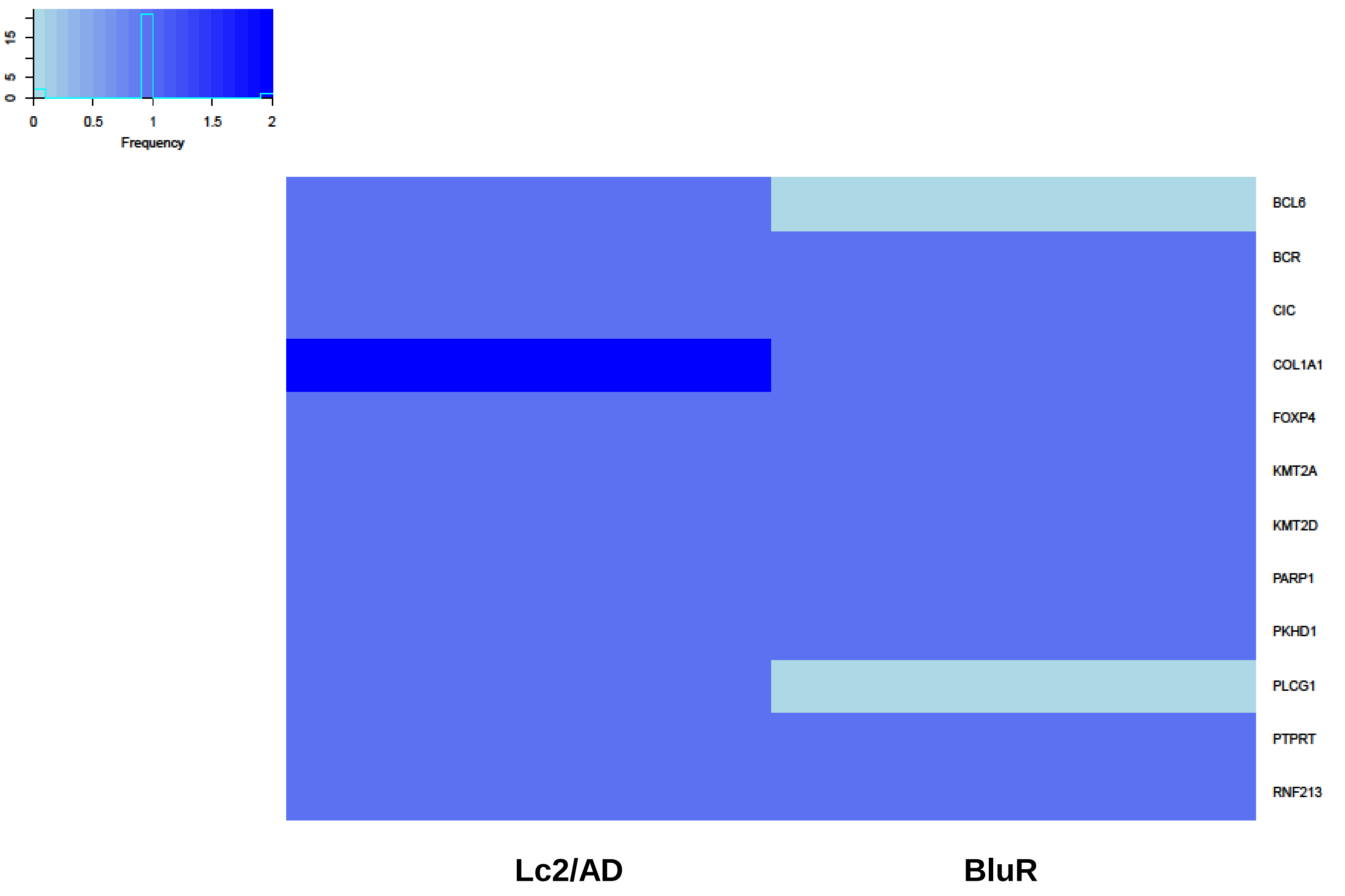

Supplementary Fig2: BluR cells did not gain new mutations. Heatmap created by RStudio software showing detected gene mutations in resistant cells (BluR) compared to baseline (LC2AD)

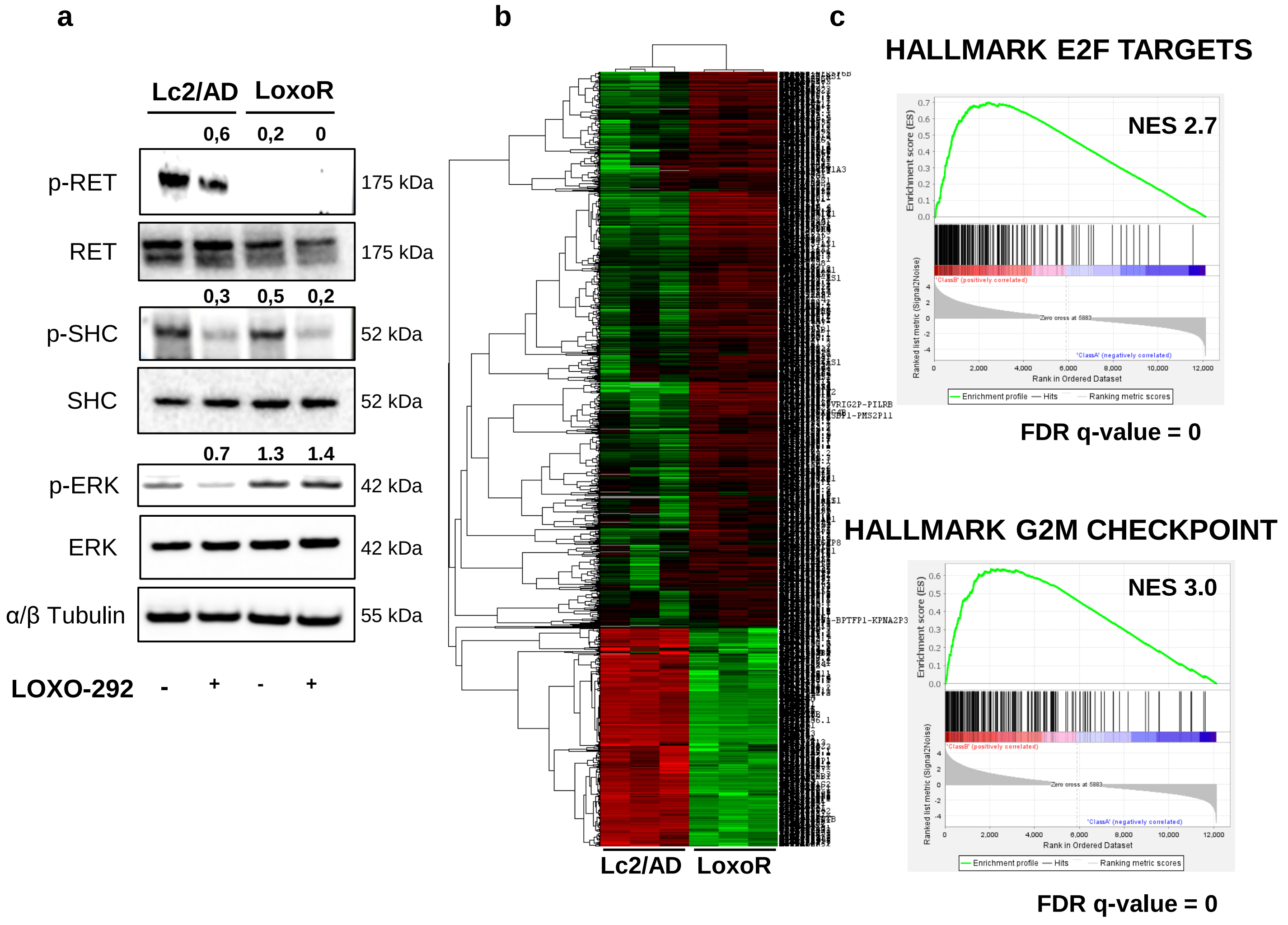

Supplementary Fig3: RNA-Sequencing of LoxoR cells shows de-regulated proliferative pathways. (a) Immunoblots of parental and LoxoR cells treated with LOXO-292 (500nM). After treatment for 4 h, whole-cell lysates were prepared and subjected to immunoblot analyses with the indicated antibodies. Numbers above blots indicate FC variations compared to vehicle-treated Lc2/AD cells. Images are representatives from three independent experiments. **(b)** Heatmap showing unsupervised hierarchical clustering of differentially expressed genes (FDR <0.01) in LoxoR versus Lc2/AD parental cells upon LOXO-292 treatment for 24h. Up- and down-regulated genes are represented in red and green, respectively. **(c)** Gene set enrichment analysis (GSEA) of significantly de-regulated pathways in LoxoR cells versus Lc2/AD cells shown with Normalized Enriched Score (NES) and FDR q-value**.**

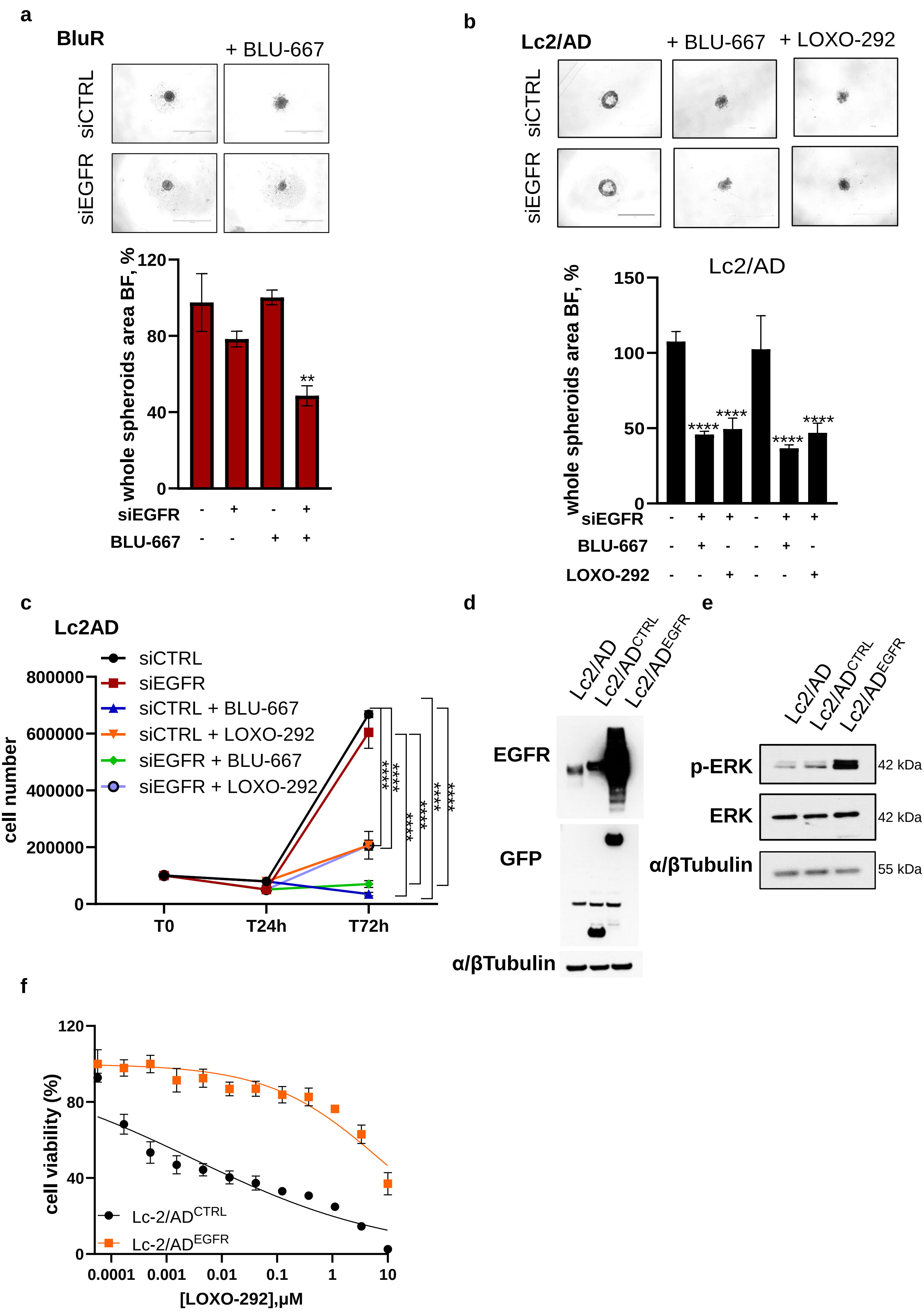

a
BluR
+ BLU-667
siCTRL
siEGFR
whole spheroids area BF, %
120
80
40
0
**
siEGFR - + - +
BLU-667 - - + +
b
Lc2/AD
+ BLU-667
+ LOXO-292
siCTRL
siEGFR
Lc2/AD
whole spheroids area BF, %
150
100
50
0
****
****
****
****
siEGFR - + + - + +
BLU-667 - + - - + -
LOXO-292 - - + - - +
c
Lc2AD
siCTRL
siEGFR
siCTRL + BLU-667
siCTRL + LOXO-292
siEGFR + BLU-667
siEGFR + LOXO-292
cell number
800000
600000
400000
200000
0
T0
T24h
T72h
****
d
Lc2/AD
Lc2/AD^CTRL
Lc2/AD^EGFR
EGFR
GFP
α/βTubulin
e
Lc2/AD
Lc2/AD^CTRL
Lc2/AD^EGFR
p-ERK
ERK
α/βTubulin
42 kDa
42 kDa
55 kDa
f
cell viability (%)
120
80
40
0
Lc-2/AD^CTRL
Lc-2/AD^EGFR
0.0001
0.001
0.01
0.1
1
10
[LOXO-292],μM

Supplementary Fig4: Lc2/AD viability and growth is EGFR signaling independent. (a-b) Representative images of BluR (a) and Lc2/AD (b) spheroids cultured for 72 hours under EGFR specific siRNAs and BLU-667 (250nM) or LOXO-292 (250nM) treatments. All images were captured at 20x magnification (Bars = 200 μm) (a,b, upper panel). The bar graph showing the whole spheroids is plotted as a percentage relative to the vehicle-treated spheroids area (a,b, bottom panel) (** $p< 0.01$; 2way ANOVA Bonferroni's multiple comparisons). **(c)** Line chart indicating cell number of Lc2/AD cells treated with scrambler small interfering RNAs (siCTRL) or siRNAs targeting EGFR ± BLU-667 and ± LOXO-292. (****$p< 0.0001$; 2way ANOVA Bonferroni's multiple comparisons). **(d-e)** Immunoblots of parental, Lc2/AD^{CTRL}, and Lc2/AD^{EGFR} cells. Whole-cell lysates were prepared and subjected to immunoblot analyses with the indicated antibodies. Images are representatives from three independent experiments. **(f)** Twelve-point dose-response curves of Lc2/AD over-expressing GFP control vector (pLenti-C-mGFP-P2A-Puro; Lc2/AD^{CTRL}, black line) or EGFR over-expressing cells (Lc2/AD^{EGFR}, orange line) treated with LOXO-292. After 6 days of treatment, cells were lysed and quantified with ATP cell titer Glo. Each data point represents the percentage of cell viability relative to vehicle-treated controls. For all panels, each point represents the mean of viability ± SD from three independent experiments.

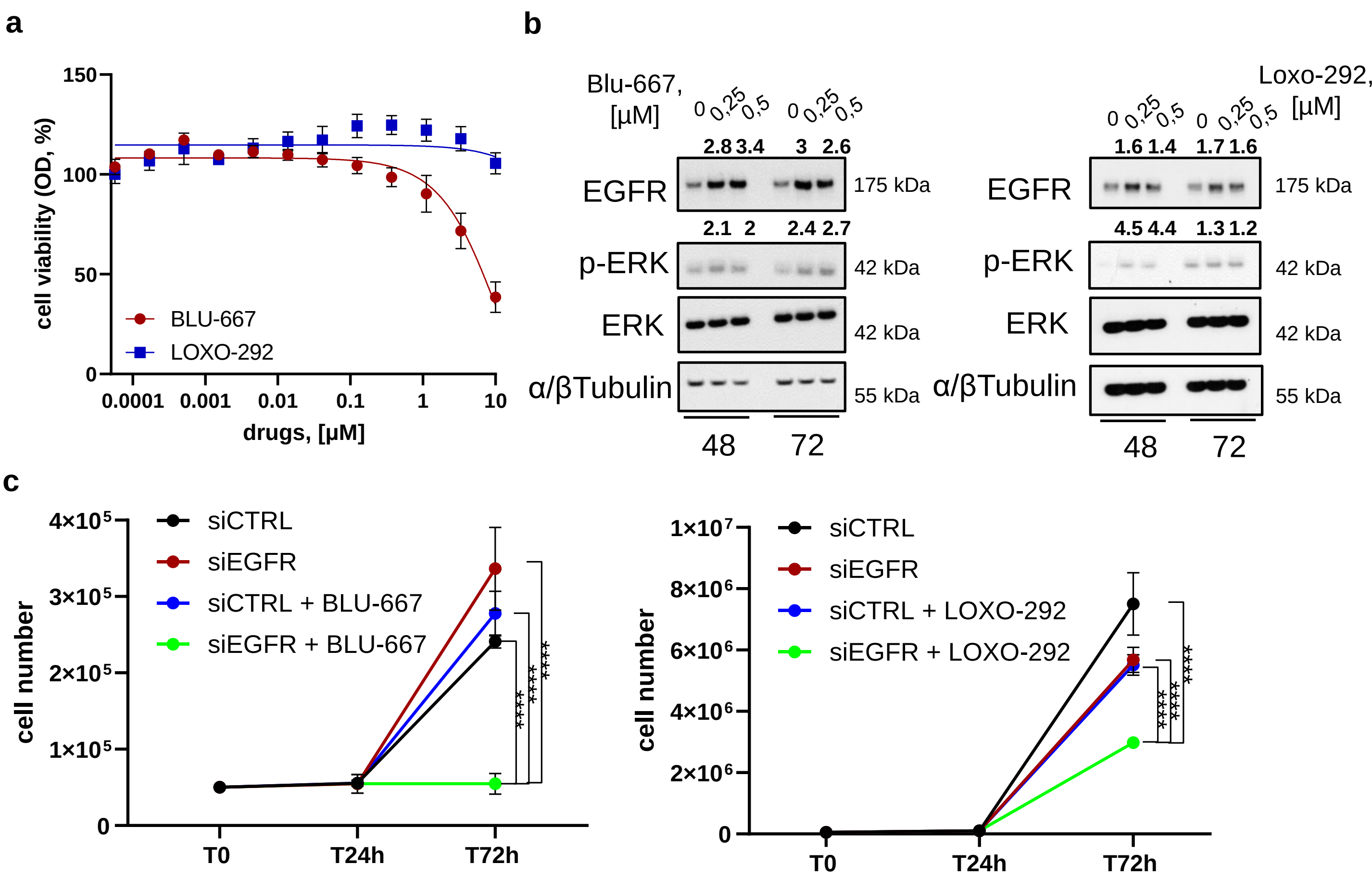

Supplementary Figure 5: papillary thiroid cancer cell line, TPC1, shows intrinsic resistance to RET inhibitors due to EGFR hyper-expression. (a) Twelve-point dose-response curves of papillary thyroid cancer cells (TPC1) treated with BLU-667 (red line) and LOXO-292 (blue line). After 6 days of treatment, cells were lysed and quantified with ATP cell titer Glo. Each data point represents the percentage of cell viability relative to vehicle-treated controls. **(b)** Immunoblots of TPC1 cells treated with increasing doses of BLU-667 and LOXO-292 (up to 500nM) for 48h and 72h. Whole-cell lysates were prepared and subjected to immunoblot analyses with the indicated antibodies. Numbers above blots indicate FC variations compared to vehicle-treated cells at each time point. Images are representatives from three independent experiments. **(c)** Line chart indicating cell number of TPC1 treated with scrambler small interfering RNAs (siCTRL) or siRNAs targeting EGFR (siEGFR) ± BLU-667 and ± LOXO-292, left and right panels respectively. (****$p < 0.0001$; 2way ANOVA Bonferroni's multiple comparison). For all panels, each point represents the mean of viability ± SD from three independent experiments.

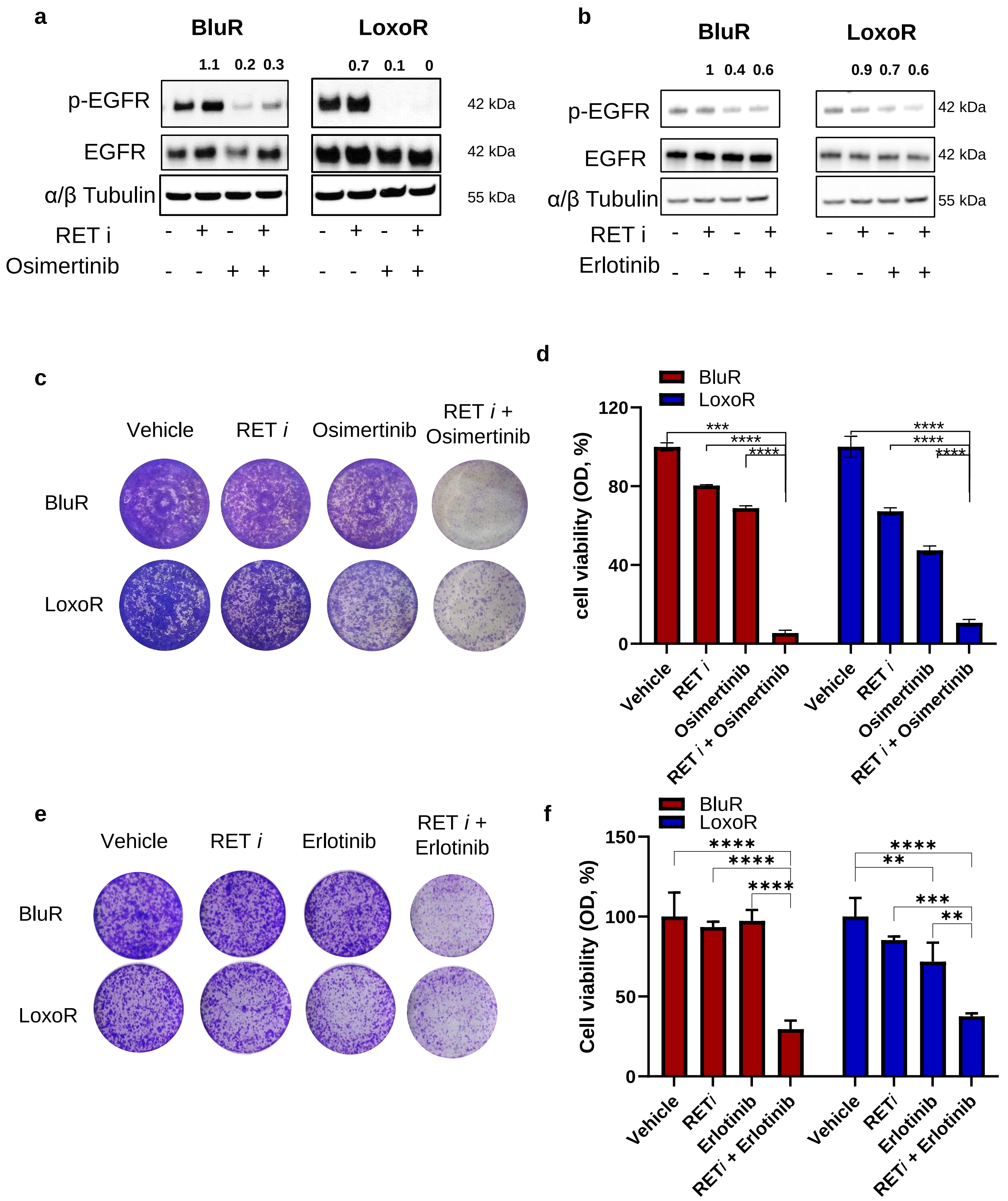

a
BluR
LoxoR
1.1 0.2 0.3
0.7 0.1 0
p-EGFR
EGFR
α/β Tubulin
42 kDa
42 kDa
55 kDa
RET i
Osimertinib
b
BluR
LoxoR
1 0.4 0.6
0.9 0.7 0.6
p-EGFR
EGFR
α/β Tubulin
42 kDa
42 kDa
55 kDa
RET i
Erlotinib
c
Vehicle
RET i
Osimertinib
RET i + Osimertinib
BluR
LoxoR
d
BluR
LoxoR
cell viability (OD, %)
120
80
40
0
***
****
****
****
****
****
Vehicle
RET i
Osimertinib
RET i + Osimertinib
Vehicle
RET i
Osimertinib
RET i + Osimertinib
e
Vehicle
RET i
Erlotinib
RET i + Erlotinib
BluR
LoxoR
f
BluR
LoxoR
Cell viability (OD, %)
150
100
50
0
****
****
****
****
**
***
**
Vehicle
RETi
Erlotinib
RETi + Erlotinib
Vehicle
RETi
Erlotinib
RETi + Erlotinib

Supplementary Figure 6. BluR and LoxoR sensitivity to osimertinib and erlotinib (a-b) Immunoblots of BluR (left) and LoxoR (right) cells treated with BLU-667 or LOXO-292 ± osimertinib (a) or ± erlotinib (b). Whole-cell lysates were prepared and subjected to immunoblot analyses with the indicated antibodies. Numbers above blots indicate FC variations compared to vehicle-treated cells. Images are representatives from three independent experiments.**(c,e)** Images of crystal violet-stained monolayers of BluR and LoxoR cells seeded in 6-well plates and treated with vehicle, BLU-667 (500nM) or LOXO-292 (500nM), osimertinib (200nM, a), erlotinib (1μM, d) and the combination of RET inhibitors *plus* EGFR inhibitors. **(d,f)** Bar graph showing quantification by spectrophotometric detection of the integrated intensity values as % of vehicle-treated controls in BluR (red) and LoxoR (blue) cells. (**p<0.01; ***p<0.001, ****p< 0.0001; two-way ANOVA)

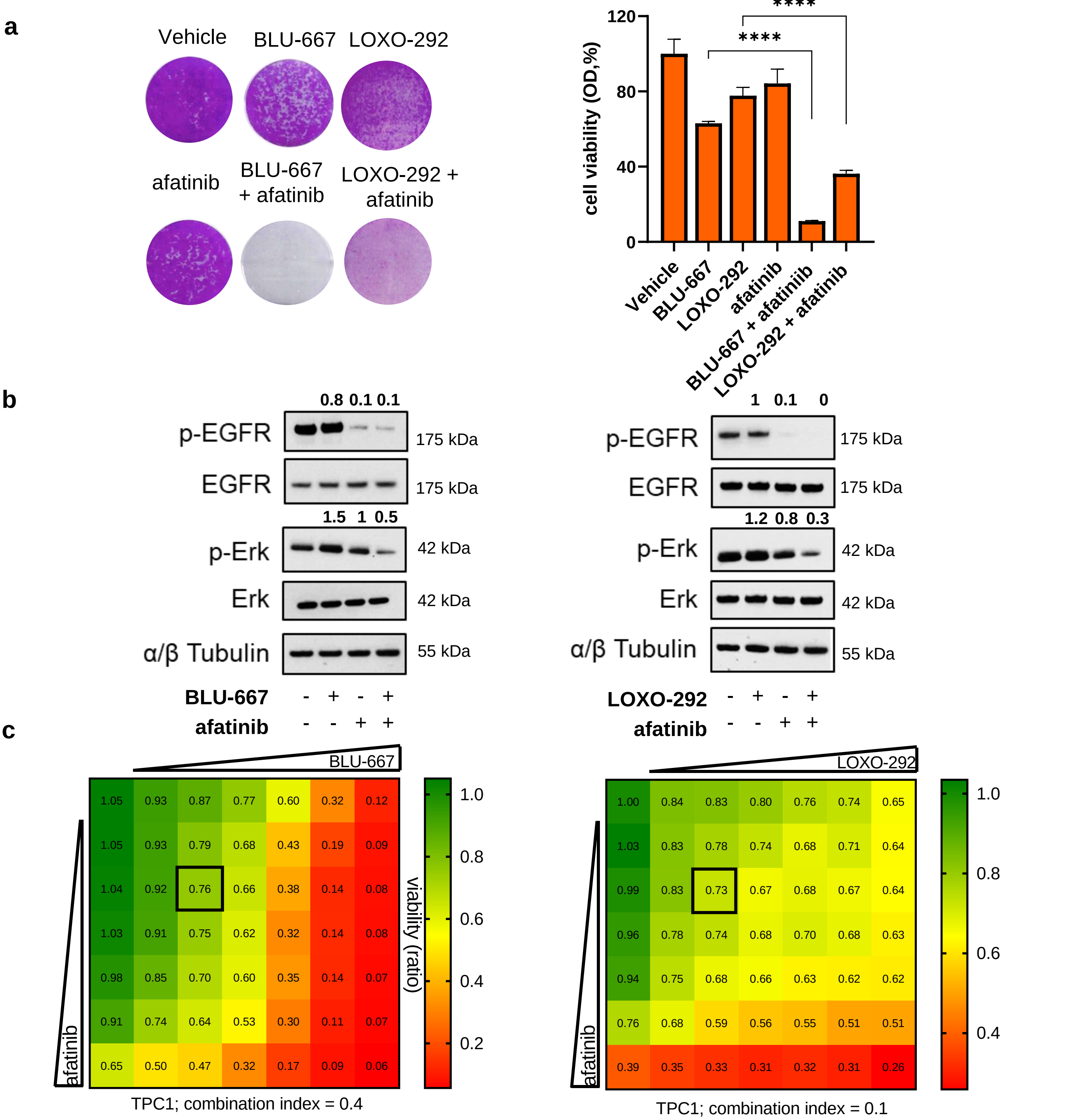

Supplementary Figure 7: RET and EGFR combination treatment re-sensitizes CCDC/6-RET TPC1 cells to BLU-667 and LOXO-292. (a) Images of crystal violet-stained monolayers of TPC1 cells seeded in 6-well plates and treated with vehicle, BLU-667 (500nM), LOXO-292 (500nM), afatinib (50nM) and the combination of RET inhibitors *plus* afatinib (left panel). Bar graph showing quantification by spectrophotometric detection of the integrated intensity values as % of vehicle-treated controls (right panel). (****$p < 0.0001$; 2way ANOVA Bonferroni's multiple comparisons). **(b)** Immunoblots of TPC1 cells treated with BLU-667 (left) or LOXO-292 (right) ± afatinib. Whole-cell lysates were prepared and subjected to immunoblot analyses with the indicated antibodies. Numbers above blots indicate FC variations compared to vehicle-treated cells. Images are representatives from three independent experiments. **(c)** Viability assay to test synergy between BLU-667 (left) or LOXO-292 (right) and afatinib in TPC1 cells. Cells were treated with increasing concentrations of each drug alone or both drugs every 72 h until vehicle-treated controls reached ~90% confluency. Cell monolayers were then lysed with ATP cell titer Glo reagent and the number of viable cells was determined based on quantification of the ATP, indicating metabolically active cells. Combination indices were determined using the Chou-Talalay test. Numbers inside each box indicate the ratio of viable treated cells to untreated cells from three independent experiments.

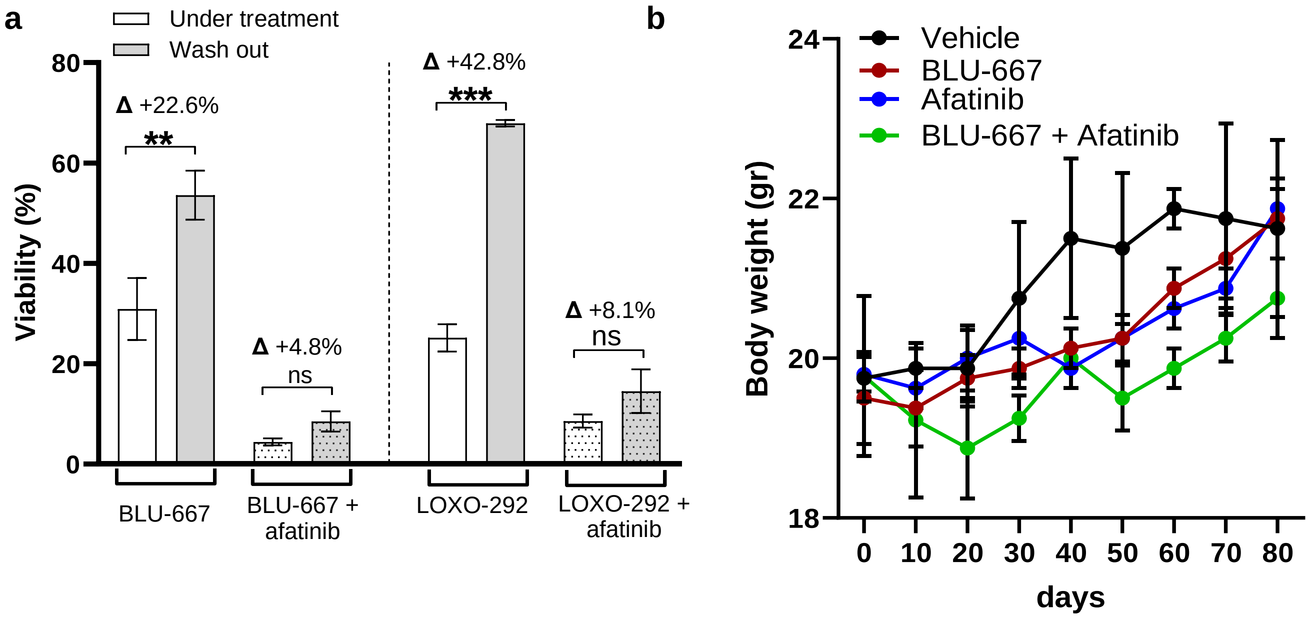

Supplementary Figure 8: Combined treatment with RET and EGFR inhibitors impairs regrowth upon drug withdrawal. (a) Bar graph showing the quantification of integrated intensity values by spectrophotometric detection, normalized to vehicle-treated controls. Lc2/AD cells were continuously treated for 14 days with vehicle, BLU-667 (500 nM), LOXO-292 (500 nM), or the indicated combinations with afatinib (50 nM), followed by a six-days washout. Data are presented as the difference (Δ) in cell viability (%) after the washout compared to viability under continuous treatment. (ns $p>0.05$; ** $p<0.01$; *** $p< 0.001$; Student's t test). **(b)** Line graph showing changes in mouse body weight after vehicle, BLU-667, afatinib, and combination therapies.

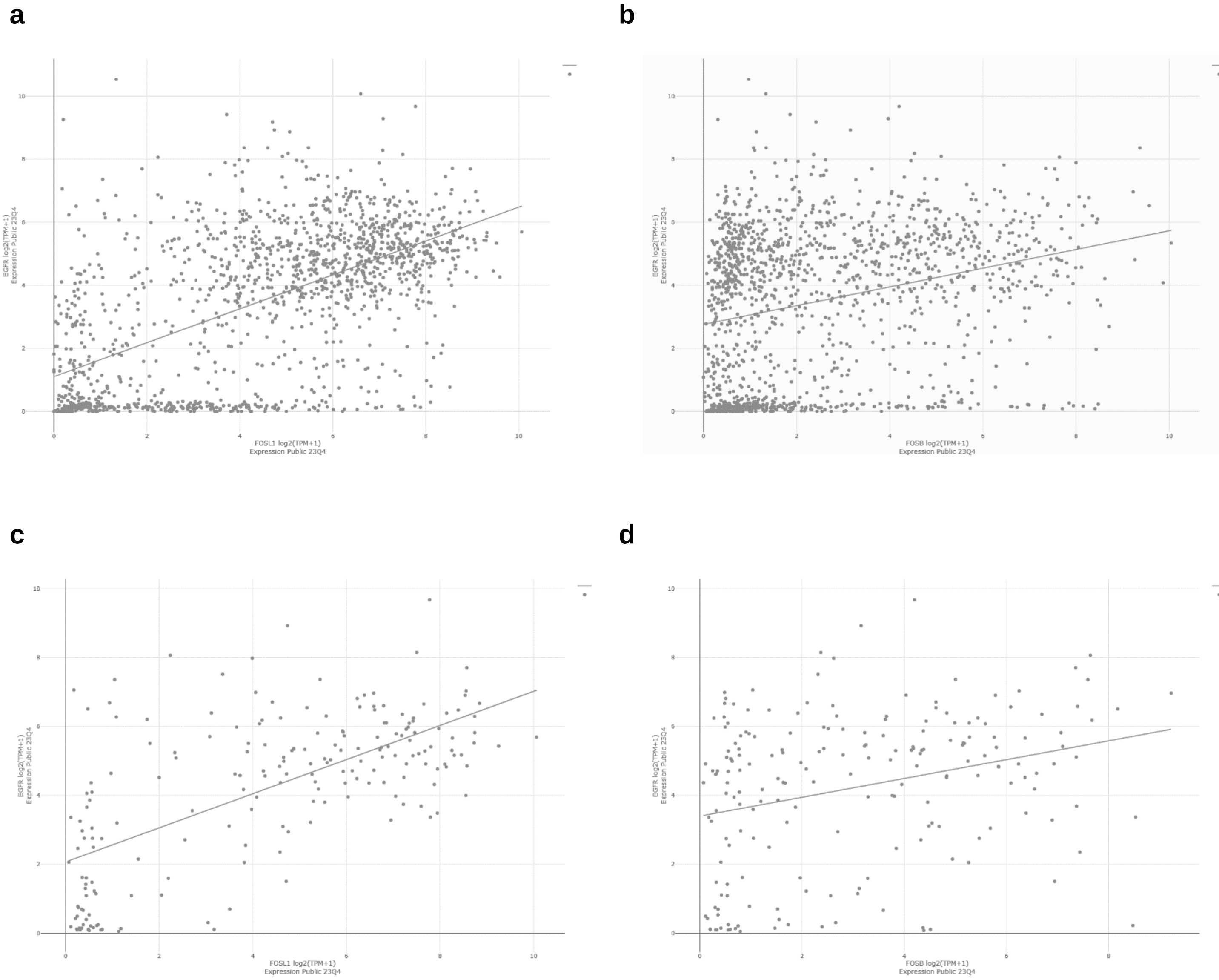

Supplementary Figure 9: DEPmap analysis reveals positive correlation between FOSL1/FOSB and EGFR expression in different tumors. (a-b) gene expression transcripts per million (TPM) values of RNA-Sequencing data are reported for FOSL1 and EGFR (a) and FOSB and EGFR (b) for all tumor cell lines in DepMap dataset 23Q4. **(c-d)** gene expression transcripts per million (TPM) values of RNA-Sequencing data are reported for FOSL1 and EGFR (c) and FOSB and EGFR (d) for lung cell lines in DepMap dataset 23Q4

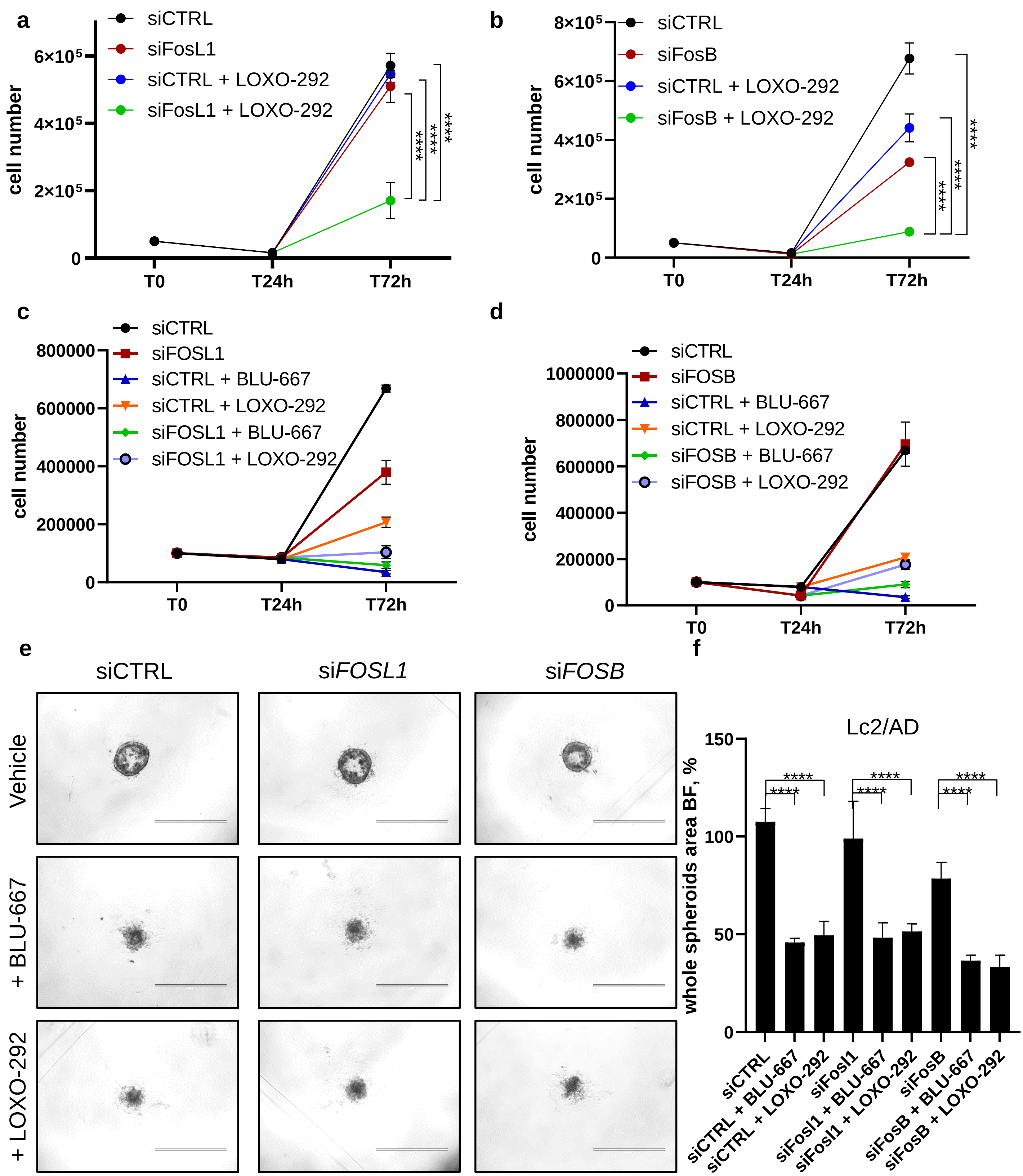

Supplementary Fig. 10: AP1 complex regulates resistance to LOXO-292 regulating EGFR expression and do not modulate Lc2/AD viability. (a-b) Line chart indicating cell number of LoxoR cells treated with scramble small interfering RNAs (siCTRL) or siRNAs targeting *FOSL1* (si*FOSL1*; a) and siRNAs targeting *FOSB* (si*FOSB*; b) ± LOXO-292. **(c-d)** Line chart indicating cell number of Lc2/AD cells treated with scramble small interfering RNAs (siCTRL) or siRNAs targeting *FOSL1* (si*FOSL1*; c) and siRNAs targeting *FOSB* (si*FOSB*; d) ± BLU-667 and ± LOXO-292. **(e)** Representative images of Lc2/AD spheroids cultured for 72 hours under *FOSL1* and *FOSB* specific siRNAs and BLU-667 (250nM) and LOXO-292 (250nM) treatments. All images were captured at 20x magnification (Bars = 200 µm). **(f)** Bar graph showing the whole spheroids is plotted as a percentage relative to of vehicle-treated spheroids area. For all panels, ****p< 0.0001; 2way ANOVA Bonferroni's multiple comparisons
